# Regulation of titin-based cardiac stiffness by unfolded domain oxidation (UnDOx)

**DOI:** 10.1073/pnas.2004900117

**Published:** 2020-09-14

**Authors:** Christine M. Loescher, Martin Breitkreuz, Yong Li, Alexander Nickel, Andreas Unger, Alexander Dietl, Andreas Schmidt, Belal A. Mohamed, Sebastian Kötter, Joachim P. Schmitt, Marcus Krüger, Martina Krüger, Karl Toischer, Christoph Maack, Lars I. Leichert, Nazha Hamdani, Wolfgang A. Linke

**Affiliations:** ^a^Institute of Physiology II, University of Munster, 48149 Munster, Germany;; ^b^Institute of Physiology, Ruhr University Bochum, 44801 Bochum, Germany;; ^c^Comprehensive Heart Failure Center Wuerzburg, University Clinic Wuerzburg, 97078 Wuerzburg, Germany;; ^d^Department of Internal Medicine II, University Hospital Regensburg, 93053 Regensburg, Germany;; ^e^Institute for Genetics, University of Cologne, 50931 Cologne, Germany;; ^f^Department of Cardiology and Pneumology, University Medicine Goettingen, 37075 Goettingen, Germany;; ^g^Department of Cardiovascular Physiology, Heinrich Heine University, 40225 Düsseldorf, Germany;; ^h^Department of Pharmacology and Clinical Pharmacology, Heinrich Heine University, 40225 Düsseldorf, Germany;; ^i^Center for Molecular Medicine and Excellence Cluster "Cellular Stress Responses in Aging-Associated Diseases" (CECAD), University of Cologne, 50931 Cologne, Germany;; ^j^Institute for Biochemistry and Pathobiochemistry, Ruhr University Bochum, 44801 Bochum, Germany

**Keywords:** oxidative stress, myocardial stiffness, single-molecule measurements, proteomics, mechanics

## Abstract

The relationship between oxidative stress and cardiac stiffness is thought to involve modifications to the giant muscle protein titin, which in turn can determine the progression of heart disease. In vitro studies have shown that S-glutathionylation and disulfide bonding of titin fragments could alter the elastic properties of titin; however, whether and where titin becomes oxidized in vivo is less certain. Here we demonstrate, using multiple models of oxidative stress in conjunction with mechanical loading, that immunoglobulin domains preferentially from the distal titin spring region become oxidized in vivo through the mechanism of unfolded domain oxidation (UnDOx). Via oxidation type-specific modification of titin, UnDOx modulates human cardiomyocyte passive force bidirectionally. UnDOx also enhances titin phosphorylation and, importantly, promotes nonconstitutive folding and aggregation of unfolded domains. We propose a mechanism whereby UnDOx enables the controlled homotypic interactions within the distal titin spring to stabilize this segment and regulate myocardial passive stiffness.

## Significance

Titin oxidation alters titin stiffness, which greatly contributes to overall myocardial stiffness. This stiffness is frequently increased in heart disease, such as diastolic heart failure. We have quantified the degree of oxidative titin changes in several murine heart and skeletal muscle models exposed to oxidant stress and mechanical load. Importantly, strain enhances in vivo oxidation of titin in the elastic region, but not the inextensible segment. The functional consequences include oxidation type-dependent effects on cardiomyocyte stiffness, titin-domain folding, phosphorylation, and inter-titin interactions. Thus, oxidative modifications stabilize the titin spring in a dynamic and reversible manner and help propagate changes in titin-based myocardial stiffness. Our findings pave the way for interventions that target the pathological stiffness of titin in disease.

Oxidative stress is an integral part of physiological processes characterized by an increased amount of reactive oxygen/nitrogen species (ROS/RNS) or a reduction in antioxidant defense mechanisms. At the more extreme level, oxidative stress can cause DNA, lipid, and protein damage leading to eventual cell death. However, at physiological levels, oxidative stress does not necessarily have harmful effects and could instead play a regulatory role. Improvements in cardiac muscle function have been observed under oxidative conditions (1, 2). Interestingly, oxidative modifications also modulate the mechanical properties of striated muscle, including the passive stiffness of the cardiomyocytes (3, 4). The elastic titin filament is of particular interest in this context, as this giant sarcomeric protein is a main contributor of passive stiffness in the myocardium and has previously been shown to be modulated in its mechanical behavior by oxidative modifications (5, 6). However, the influence of oxidative stress on the redox state of titin has mainly been studied in a few selected titin regions in vitro and inferred in isolated cell preparations. Uncertainty exists about where relevant oxidation of titin occurs in vivo and what functional role it may play.

The elasticity of titin is made possible through the flexible I-band portion in the sarcomeres, the smallest structural units of striated muscles. In contrast, the titin segments in other sarcomeric regions (Z-disk, A-band, and M-band) are largely inelastic, because they are bound to relatively stiff protein complexes. The main structural components of I-band titin are the numerous evolutionarily well-conserved tandem-immunoglobulin-like (Ig) domains. These I-band Ig domains contain on average two cysteines, which are potentially available for reversible oxidative modification, such as S-glutathionylation or disulfide (S-S) bonding (6–8). In contrast, the domains located within A-band titin contain on average only one cysteine (6) whose function is obscure. Importantly, many cysteines in I-band Ig domains of titin appear to be “cryptic,” i.e., they are only accessible for redox modification when the domain is in the unfolded state (6). Unfolding and refolding does take place in the Ig domains from elastic titin at physiological sarcomere lengths (SLs) and low stretch forces (9, 10), contributing to alterations in titin-based myocardial stiffness (10). If unfolded, the titin Ig domains become S-glutathionylated in the presence of oxidized glutathione (GSSG), which then prevents the folding of the domain back to its native conformation (6). Because S-glutathionylation weakens the mechanical stability of the Ig domain, it lowers the titin-based stiffness of cardiomyocytes when they are exposed to GSSG in a stretched state (6). This effect on stiffness is reversed when reducing conditions are introduced.

In addition to S-glutathionylation, oxidative stress can cause intramolecular S-S bonding between specific pairs of cysteines within Ig domains of I-band titin, which prevents the complete unfolding of the Ig domain (8). This modification could, in theory, increase titin stiffness. If there are more than two accessible cysteines present, like in many Ig domains from the middle (differentially spliced) I-band titin segment, disulfide isomerization may occur (8). Interestingly, the enzyme protein disulfide isomerase (PDI) promotes disulfide bonding in recombinant titin Ig domains (11). If unfolded, titin Ig domains are able to refold more rapidly in the presence of oxidized PDI through the formation of S-S bonds (11). Conversely, the disulfide-bonded but folded Ig domain unfolds at lower stretch forces than the reduced, folded Ig domain (11). How disulfide bonding within the Ig domains alters titin-based stiffness in muscle cells has not been measured. Moreover, it is unresolved whether (and where in the molecule) changes in titin oxidation state occur if oxidative stress is induced in the heart. Understanding both in vivo and in vitro effects of titin oxidation would allow us to make predications as to whether oxidative stress plays a regulatory role in modulating titin stiffness of healthy and diseased hearts.

Here, we set out to determine where titin is oxidized in situ/in vivo, with the main aim being to test the hypothesis that sarcomere stretching in the presence of oxidant stress increases titin oxidation more in the elastic I-band than in the inextensible A-band. Further, we elucidate how titin-based stiffness of human cardiomyocytes is altered by S-glutathionylation vs. S-S bonding, and we evaluate the importance of stretch in enabling these processes to occur. We find that stretch promotes oxidation of cysteines in unfolded Ig domains of titin in situ, a process we coin UnDOx (unfolded domain oxidation). Strikingly, UnDOx consistently occurs in Ig domains of the distal I-band titin segment, which has been thought to be largely inert to Ig-domain unfolding. Based on mechanistic insight gained from our mechanical measurements on isolated human cardiomyocytes, in vitro analyses of wild-type (WT) and mutant proteins, and single-molecule atomic force spectroscopy, we propose a scenario whereby UnDOx regulates the controlled (in-register) aggregation of distal I-band Ig domains. This mechanism can stabilize the distal titin spring region in a dynamic and reversible manner and help synchronize and propagate changes in titin-based passive tension of the heart.

## Results

### Ex Vivo H_2_O_2_ Elevates Oxidation of Titin and Other Cardiac Proteins.

We measured the ex vivo or in vivo redox state of titin in different murine heart and skeletal muscle models, in which oxidative stress was induced. To this end, we used the OxICAT method coupled with mass spectrometry (MS) (12). OxICAT involves using a cysteine-specific isotope-coded affinity tag (ICAT) reagent to differentially label oxidized and reduced cysteines, which can then be detected with MS. By systematically labeling all reduced cysteines in the tissue sample first and then labeling the remaining cysteines that were in an oxidized state in the tissue, we were able to minimize any artificial oxidation from occurring and reliably quantify changes in redox state between our models of oxidative stress and control conditions.

**Fig. 1.**
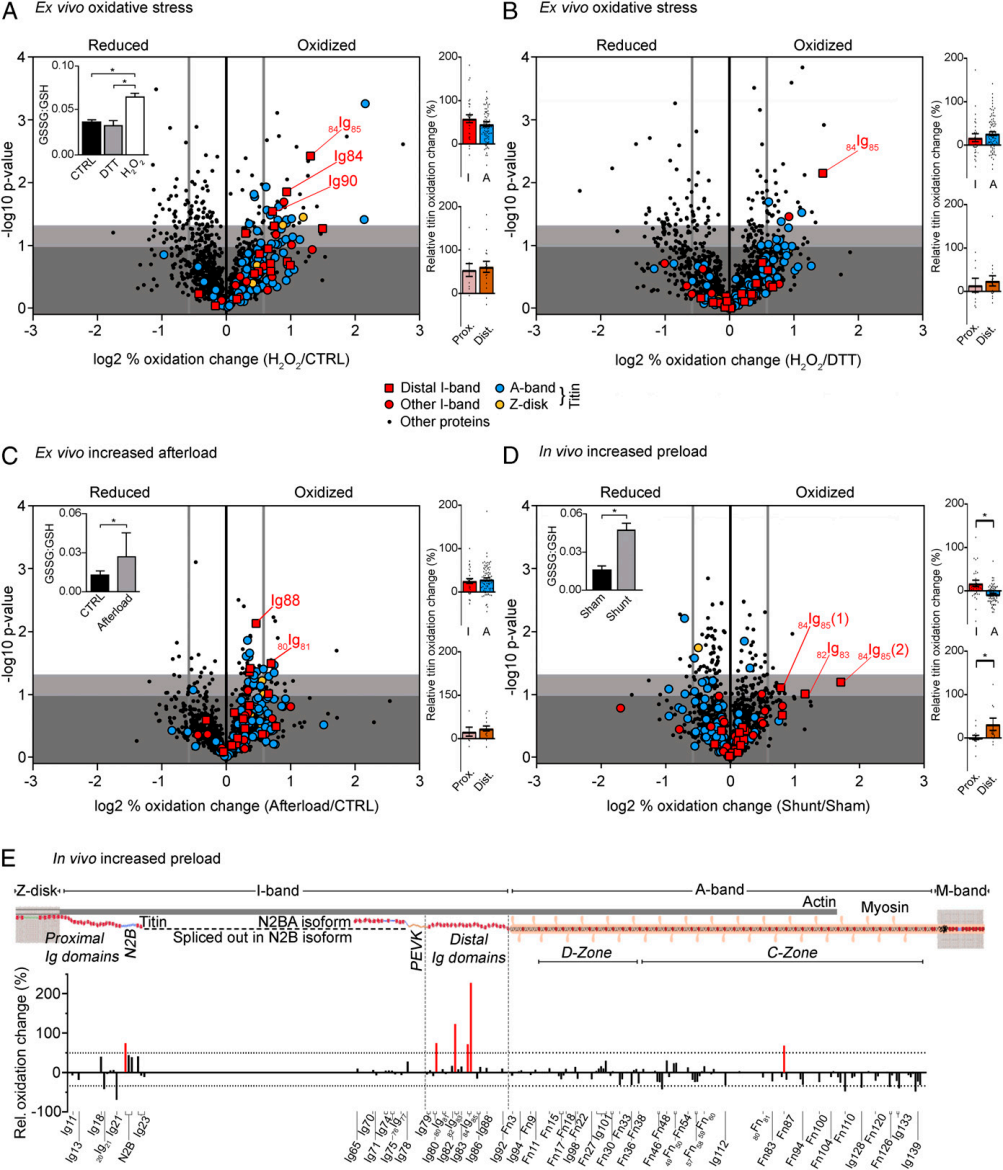
Redox state of cardiac proteins in different mouse heart models. Volcano plots of peptides identified in the Langendorff H_2_O_2_ perfusion (*n* = 3) vs. control (*n* = 3) (*A*) or DTT (*n* = 3) (*B*), afterload-increase heart model (*n* = 4 for control and afterload increase) (*C*), and in vivo shunt (*n* = 4) vs. sham (*n* = 3) model (*D*). The difference in (log2) percentage oxidized was used. A threshold value of +0.58/−0.58 was chosen for redox classification (thick vertical gray line). Nonsignificant changes, dark gray background; sites trending toward significances between −log10 1.00 to 1.30, light gray background; and significantly altered sites above −log10 of 1.30, white background. *Insets*: GSSG:GSH ratio for H_2_O_2_ Langendorff perfusion (*A*; *n* = 6), afterload increase (*C*; *n* = 9) and shunt vs. sham (*D*; *n* = 4). Colored bar graphs on *Right*: percentage oxidation increase in I-band vs. A-band titin (*Top*) and proximal vs. distal I-band titin (*Bottom*). Data are means ± SEM *P < 0.05, two-tailed Mann–Whitney *U* test. For data in *Insets*, significance was tested by ANOVA, followed by Tukey’s test (*A*) or unpaired, two-tailed Student’s *t* test (*C* and *D*). (*E*) Schematic of mouse cardiac titin and bar graph showing the percentage oxidation change against the associated titin domains (UniProtKB ID A2ASS6). Increases by >50% are highlighted in red.

Initially, to induce oxidative stress ex vivo, a Langendorff perfusion (retrograde through the aorta) of isolated mouse hearts was performed as a proof-of-principle study using 0.1% (vol/vol) H_2_O_2_ in physiological saline. This high H_2_O_2_ concentration was chosen aiming to maximize the number of oxidized cardiac peptides. As a control, other hearts were perfused with pure physiological saline (CTRL) or 1 mM 1,4-dithiothreitol (DTT, a reductant) in physiological saline. Left ventricular (LV) samples were processed using the ICAT reagent and submitted to MS to identify differences in redox state of the cardiac proteome. Increased oxidative stress was confirmed by measuring the GSSG to reduced glutathione (GSH) ratio, which changed from 0.038 ± 0.002 and 0.033 ± 0.006 in CTRL and DTT-perfused samples, respectively, to 0.065 ± 0.005 in H_2_O_2_-perfused hearts (mean ± SEM; Fig. 1 *A*, *Inset*). Using MS, we were able to detect and compare 1,327 different peptides in H_2_O_2_ vs. CTRL samples, and found an increase in oxidation in ∼10% of the peptides leading to an overall 4% relative increase in proteomic oxidation (Fig. 1*A* and *SI Appendix*, Table S1). Many of the peptides frequently oxidized were sarcomere specific, including titin, in addition to various mitochondrial peptides (*SI Appendix*, Fig. S1). A closer look at titin revealed that almost half of the 125 peptides detected showed increased oxidation (49 ± 5%) and were relatively evenly distributed between I-band and A-band segments (Fig. 1 *A*, *Top Right* and *SI Appendix*, Table S2). When H_2_O_2_ was compared to DTT perfusion, a total of 1,338 peptides were detected and the whole proteome oxidation increased by 7 ± 1% (Fig. 1*B* and *SI Appendix*, Table S1). Again, the increase in titin oxidation (117 peptides detected) was relatively higher (23 ± 4%), with I-band and A-band titin regions being similarly affected (Fig. 1 *B*, *Top Right* and *SI Appendix*, Table S2). There was also no significant difference in the mean relative oxidation change of proximal vs. distal I-band titin peptides (Fig. 1 *A* and *B*, *Bottom Right* and *SI Appendix*, Table S2). We conclude that cardiac titin oxidation greatly increases under conditions of ex vivo oxidative stress, particularly when compared with the entire cardiac proteome.

### Increased Afterload Promotes Oxidation of Titin and Other Cardiac Proteins.

In the Langendorff-perfused hearts, oxidative stress was directly induced by perfusion with high concentrations of H_2_O_2_, which is but one of many types of ROS. Therefore, an alternative system was sought which simulated the systemic circulation for a longer time and simultaneously caused oxidative stress in a more physiological way. The ex vivo working-heart mouse model allows the heart to continuously beat throughout the perfusion, while the mechanical load can be readily adjusted. Increases in afterload, which mimic a central feature of various cardiovascular diseases, induce oxidative stress through mitochondrial ROS production (13). Thus, we increased the afterload of the working-heart model from 80 mmHg (CTRL) to 120 mmHg (afterload). Increased oxidation did occur under elevated afterload vs. control conditions, as supported by the raised GSSG:GSH ratio (Fig. 1 *C*, *Inset*), which has also been previously reported for this model (13). Following ICAT-labeling and MS, we were able to detect a total of 1,214 different peptides, 133 of which were titin peptides. The whole-proteome and titin-oxidation increases in high-afterload vs. CTRL hearts, 6 ± 1% and 29 ± 3% (mean ± SEM), respectively, were reminiscent of those observed in the Langendorff-perfusion hearts (Fig. 1*C* and *SI Appendix*, Table S1). Moreover, the distribution of oxidized peptides was again similar in I-band and A-band titin and between proximal and distal I-band titin (Fig. 1 *C*, *Right* and *SI Appendix*, Table S2). Thus, ex vivo afterload increase can promote elevated cardiac protein oxidation, especially along the entire titin molecule.

### Differential Titin Oxidation Changes Are Detected with In Vivo Preload Increase.

Next, we set out to determine whether we could detect redox modifications of cardiac proteins in vivo, including titin modifications, under conditions promoting cardiomyocyte strain. For this purpose, we used the mouse aortocaval shunt model, which we developed previously (14, 15). The induction of an aortocaval shunt between the abdominal aorta and inferior vena cava results in a large (twofold) elevation in end-diastolic pressure and volume (preload increase) after only 6 h, increases cardiomyocyte length, and triggers superoxide production to induce oxidative stress in the heart (14, 15). Using ICAT labeling of heart tissue followed by MS, the level of cardiac protein oxidation detected from shunt animals was compared with sham-operated animals. A total of 1,234 different peptides were identified in these hearts, including 127 titin peptides (Fig. 1*D* and *SI Appendix*, Table S1). In shunt vs. sham samples, increased cardiac peptide oxidation occurred but was not as prominent as in the previous models, although a significant increase in the GSSG:GSH ratio was still detected (Fig. 1 *D*, *Inset*). For the whole cardiac proteome, a 2.2 ± 0.6% rise in oxidation was seen, whereas for titin, the increase was 0.6 ± 3.0% (mean ± SEM; *SI Appendix*, Table S1). Importantly, I-band titin peptides were significantly more oxidized than A-band peptides (17 ± 8% vs. −8 ± 2%, i.e., decreased oxidation in A-band titin; Fig. 1 *D*, *Top Right*). A closer look at the I-band titin peptides revealed that Ig domains in the distal segment showed significantly higher oxidation increases compared with the proximal region (31 ± 14% vs. 1 ± 5; Fig. 1 *D*, *Bottom Right* and *SI Appendix*, Table S2) in shunt vs. sham hearts (Fig. 1 *D* and *E*), substantiating a trend already seen in the other heart models (Fig. 1 *A*–*C*). Only a few cysteines were detected in the middle (differentially spliced) I-band region, because this region is spliced out in the N2B-titin isoform that dominates in adult mouse hearts (Fig. 1*E*). The preferential oxidation increase of the distal titin spring segment became even more clear when focusing on individual Ig domains, with the largest increases seen in _84_Ig_85_ (227%; cysteine 2) and _82_Ig_83_ (123%; cysteine 1) (Fig. 1*E*). Note that our domain numbering refers to the UniProtKB ID A2ASS6, the consensus sequence of mouse titin, which does not annotate all known Ig domains of titin as such domains. For instance, _82_Ig_83_ is an Ig domain—also known as I27 or I91 (16, 17)—which is still wrongly annotated in the database as an intervening sequence between Ig domains Ig82 and Ig83. In summary, the observed pattern of differential titin oxidation suggested that the chronic preload increase promoted cardiomyocyte stretching and I-band titin lengthening by domain extension and unfolding, thereby raising Ig-domain oxidation, which mainly affected the distal titin springs.

### Strain Promotes Stronger Oxidation of Elastic vs. Inextensible Titin Regions.

Next, we aimed to directly test whether increased sarcomere strain in the presence of oxidant stress induces UnDOx and differential oxidation between I-band and A-band titin. For these experiments, we used whole mouse skeletal muscles, in order to control the stretching more accurately than in the heart models. To achieve extension of the sarcomeres, leg muscles (vastus lateralis) were stretched ex vivo by ∼20% and oxidative stress was induced using the strong oxidizing mixture of 2 mM GSH and 0.5 mM diamide. Given the consistent increase in the GSSG:GSH ratio seen in all cardiac models tested (Fig. 1, *Insets*), this oxidation combination was chosen, as it allows for more specific S-glutathionylation (and not other forms of oxidative modification that can arise using other oxidizing agents) and ensures optimal S-glutathionylation of proteins by preventing GSH depletion (18). As a control, nonstretched leg muscles were used.

**Fig. 2.**
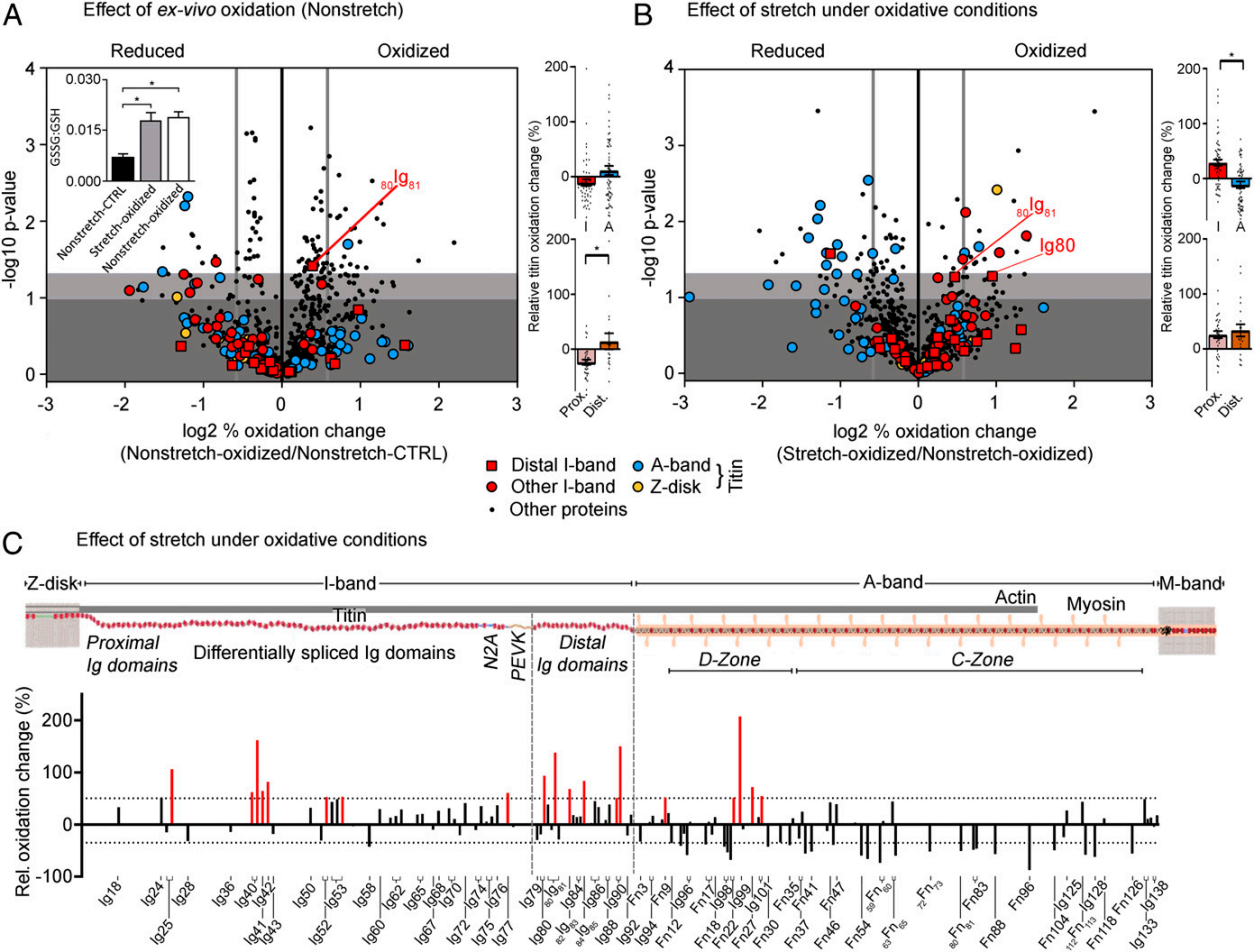
Redox state of the skeletal muscle. Volcano plots of peptides identified in nonstretched oxidized (*n* = 3) vs. nonstretched control muscle (*n* = 3) (*A*) and nonstretched oxidized (*n* = 3) vs. stretched oxidized muscle (*n* = 3) (*B*). Oxidized samples were incubated in relaxing buffer containing 2 mM GSH/0.5 mM diamide. The difference in (log2) percentage oxidized was used. A threshold value of +0.58/−0.58 was chosen (thick vertical gray line) for redox classification. Nonsignificant changes, dark gray background; sites trending toward significances between −log10 1.00 and 1.30, light gray background; and significantly altered sites above −log10 of 1.30, white background. *Inset* in *A*, GSSG:GSH ratio (*n* = 6). Colored bar graphs on *Right*: percentage oxidation increase in I-band vs. A-band titin (*Top*) and proximal vs. distal I-band titin (*Bottom*). Data are means ± SEM *P < 0.05, two-tailed Mann–Whitney *U* test. For data in *Inset* (*A*), significance was tested by ANOVA, followed by Tukey’s test. (*C*) Schematic of mouse skeletal muscle titin and bar graph showing the percentage oxidation change against the associated titin domains (UniProtKB ID A2ASS6). Increases by >50% are highlighted in red.

To establish the effects of the GSH and diamide alone, nonstretched muscles in the absence and presence of the oxidizing mixture were compared using ICAT-labeling and MS. For the 733 total peptides detected, an 11 ± 2% (mean ± SEM) relative increase in oxidation was observed, higher than what was found in the heart models (Fig. 2*A* and *SI Appendix*, Table S1). A significant increase in the GSSG:GSH ratio was also seen in oxidized vs. control muscle (0.019 ± 0.002 vs. 0.007 ± 0.001; Fig. 2 *A*, *Inset*). Interestingly, the oxidation changes in titin included relatively higher A-band oxidation and relatively lower I-band oxidation; however, the distal I-band titin segment still showed significantly increased oxidation compared to the proximal segment (Fig. 2 *A*, *Right* and *SI Appendix*, Table S2). Then, to determine the effect of stretch itself on protein oxidation, nonstretched and stretched muscle that had been incubated with GSH and diamide were compared (Fig. 2*B*). We were able to detect 686 peptides, with a whole proteome oxidation increase of 6 ± 2%, including 127 titin peptides with an increased oxidation of 9 ± 4% (*SI Appendix*, Table S1). I-band titin but not A-band titin was significantly more oxidized, and the trend of increased oxidation in distal vs. proximal I-band titin was maintained (Fig. 2 *B*, *Right* and 2*C* and *SI Appendix*, Table S2). Several more strongly oxidized cysteines were detected in the middle I-band Ig domains, which are spliced-in in the skeletal muscle titin (N2A) isoforms (Fig. 2*C*). Collectively, our findings establish that titin oxidation occurs in vivo and rises under oxidant stress, and that sarcomere stretch is an important factor in determining where titin becomes oxidized.

### Oxidation Type Determines the Direction of Stiffness Change in Stretched Cardiomyocytes.

**Fig. 3.**
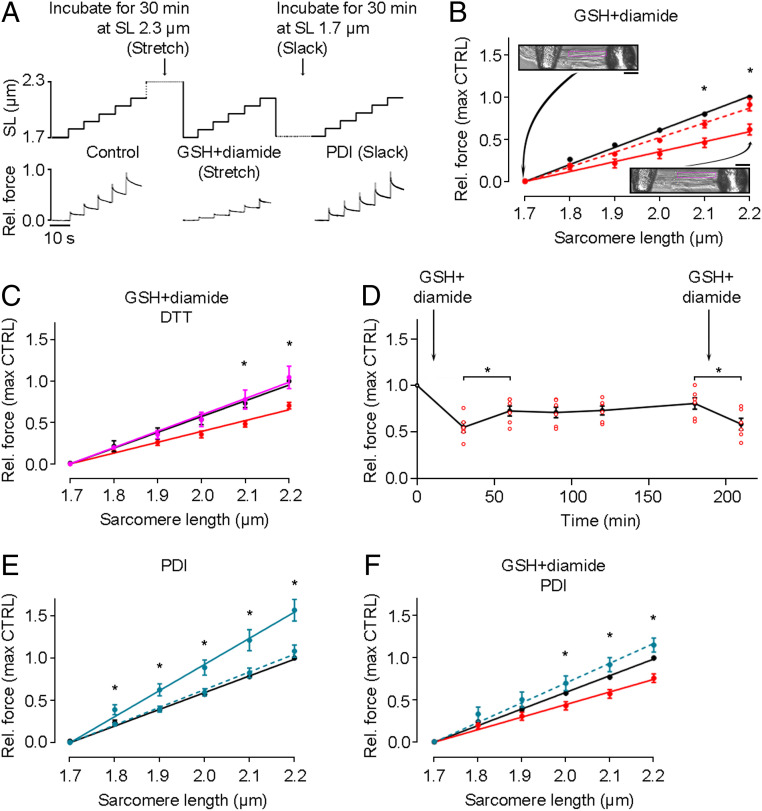
Effects of S-glutathionylation and disulfide bonding on passive force of human cardiomyocytes. (*A*) Stepwise stretch protocols used on skinned cardiomyocytes and example force traces. First, a control stretch protocol was conducted, followed by a 30-min incubation at either stretched or slack length in the absence or presence of 2 mM GSH/0.5 mM diamide or 35 µM PDI. (*B*) Passive force measured under control conditions (black), followed by incubation with 2 mM GSH/0.5 mM diamide at stretched length (solid red line, *n* = 5) or slack (dashed line, *n* = 3). *Insets* show images of a permeabilized cell attached to needles at slack (*Top*) and after stretch (*Bottom*). (Scale bars, 10 µm.) (*C*) Passive force measured under control conditions (black) followed by incubation with 2 mM GSH/0.5 mM diamide at stretched length (red), then another 30-min incubation with 5 mM DTT at stretched length (magenta) (*n* = 5). (*D*) Change of passive force with incubation of 2 mM GSH/0.5 mM diamide at stretched length and (after return to slack length) periodically remeasured before the oxidant was reintroduced (*n* = 6 cells; note that some data points overlay each other). (*E*) Passive force measured under control conditions (black), followed by incubation with 35 µM PDI at stretched length (solid blue line, *n* = 3) or slack (dashed line, *n* = 4). (*F*) Passive force measured under control conditions (black), followed by incubation with 2 mM GSH/0.5 mM diamide at stretched length (red), then another 30-min incubation with 35 µM PDI at slack length (blue) (*n* = 7). Cardiomyocytes were from a human donor heart. Peak force measured at the end of each stretch step was normalized to the control value at SL 2.2 µm. Data are means ± SEM; fits are linear regressions; *P < 0.05, by unpaired, two-tailed Student’s *t* test.

We then set out to measure the mechanical consequences of oxidation and stretch. Earlier, we showed that the passive force of skinned human cardiomyocytes became greatly reduced when the cells were incubated with 10 mM GSSG for 30 min in an overstretched state (SL, 2.6 to 2.7 µm) (6). Here, we performed a stepwise stretch protocol (SL, 1.7 to 2.2 µm) using single skinned human cardiomyocytes that were initially incubated with 2 mM GSH and 0.5 mM diamide in relaxing buffer at either a SL of 2.3 µm (stretch) or 1.7 µm (slack); i.e., at the high and low ends of the physiological SL range, respectively (Fig. 3 *A* and *B*). Passive force in the subsequent stepwise-stretch recordings was found to be significantly lower when GSH and diamide had been incubated at stretched length compared with slack length (Fig. 3*B*). For example, at 2.1 and 2.2 µm SL, passive force was reduced to ∼60% of the control value (solid red line), whereas little reduction was seen in cells previously incubated at slack length (dashed red line). The reduction in passive force with GSH+diamide treatment was reversed after incubation with 5 mM DTT in the stretched state (Fig. 3*C*, magenta line). Interestingly, this reduction was only partially maintained over the course of 3 h following treatment with GSH+diamide, but it could be reestablished with another addition of GSH+diamide (Fig. 3*D*).

The oxidant mixture of GSH and diamide specifically promotes S-glutathionylation. However, S-S bond formation between cryptic cysteines in titin Ig domains has also been suggested as a titin modification under oxidative stress and has been shown to prevent the complete unfolding of the Ig domains in vitro (8, 11). Whether S-S bonding in titin Ig domains alters the titin-based passive stiffness of cardiomyocytes has not been measured. To address this important point, demembranated single human cardiomyocytes were incubated with an oxidized form of the PDI enzyme at both slack length (SL 1.7 µm) and at stretched length (SL 2.3 µm) for 30 min and subsequently, the passive force was measured in the stepwise stretch protocol. No change in passive force was seen when the cell was incubated with PDI at slack length (Fig. 3*E*, dashed line). In contrast, when the cell was stretched during the oxidant incubation, there was a significant increase in passive force. At all SLs above slack (1.8 to 2.2 µm), passive force was significantly higher when the cells had been incubated with PDI at stretched length compared with slack incubation equivalents; e.g., passive force after PDI was ∼140% of the control measurement at SL 2.2 µm (Fig. 3*E*, solid blue line). This again indicates that stretch is first required for S-S bond formation to occur within titin domains, which then in turn increases passive force.

Having determined that the direction of changes in passive tension was dependent on the type of oxidation, we wanted to investigate the effect of consecutive addition of the different oxidants. S-glutathionylation was performed first at stretched length to aid in the stabilization of the unfolded titin I-band domains. Then, GSH and diamide were removed and replaced with oxidized PDI at slack length to promote disulfide bond formation. We observed an overshoot of the passive force to ∼115% of the original control value (Fig. 3*F*), and a significant increase in passive force measured after PDI incubation at SLs 2.0 to 2.2 µm when compared with the GSH+diamide incubation. These findings suggest that the two forms of reversible titin oxidative modifications can work synergistically to modulate passive force in cardiomyocytes bidirectionally.

### UnDOx Occurs in Various Distal I-Band Titin Ig Domains.

**Fig. 4.**
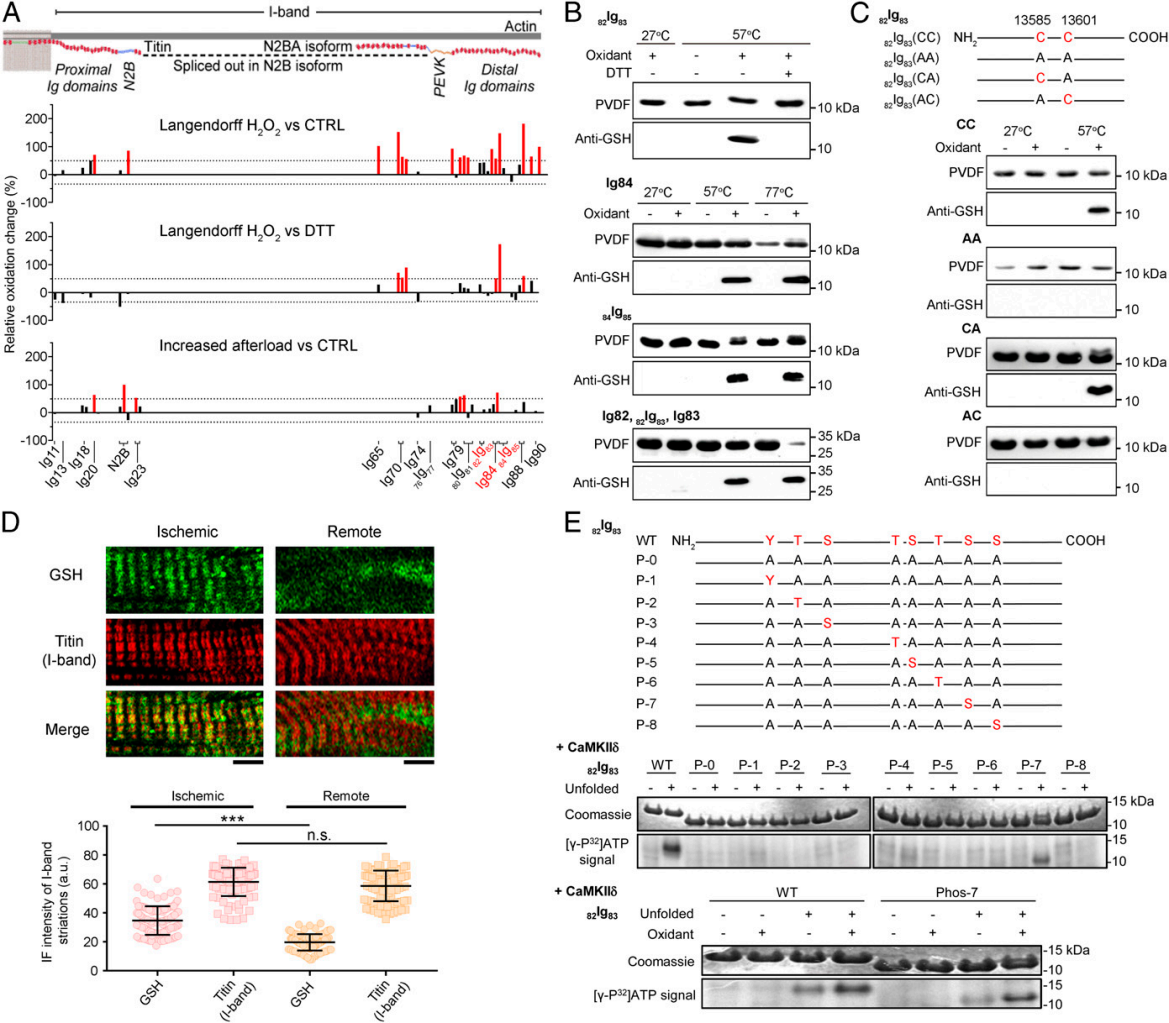
UnDOx in vitro and in vivo and unfolding-induced phosphorylation of Ig domains. (*A*) Schematic of mouse cardiac I-band titin and bar graphs showing the percentage oxidation change identified by MS against the associated titin domains (UniProtKB ID A2ASS6), for the Langendorff H_2_O_2_ vs. control (*Top*) or DTT (*Middle*) and increased afterload vs. control (*Bottom*) experiments. (*B*) Western blots (anti-GSH antibody) of recombinant Ig domains _82_Ig_83,_ Ig84, _84_Ig_85_ and three-domain construct (Ig82-_82_Ig_83_-Ig83) thermally unfolded at 27 °C, 57 °C, or 77 °C and then oxidized with 2 mM GSH/0.5 mM diamide; _82_Ig_83_ was also deglutathionylated with 5 mM DTT. (*C*) Representation of wild-type _82_Ig_83_ and cysteine (*C*) mutant constructs (*Top*) and Western blots of recombinant _82_Ig_83_ thermally unfolded at 27 °C or 57 °C and then oxidized with 2 mM GSH/0.5 mM diamide. CC, wild type; AA, both Cs replaced by alanine (*A*); CA, only C13601 replaced by A; AC, only C13585 replaced by A. (*D*) Double immunofluorescence staining of glutathione (GSH) and I-band titin in ischemic and remote heart tissue from myocardial ischemia mice (*Top*) and quantification of immunofluorescence intensity (*Bottom*). (Scale bars, 5 µm.) Data are mean ± SD; *n* = 103 (ischemic) and *n* = 93 (remote), from two hearts/group; ***P < 0.001 by ANOVA followed by Tukey’s test. n.s., non-significant. Care was taken to analyze cells of similar sarcomere length (∼2.1 µm). (*E*) Representation of _82_Ig_83_ WT and phosphomutants (*Top*). P-0, all potential phosphosites replaced by A. P-1 to P-8, mutant constructs containing only one potential phosphosite. *Bottom* shows CaMKIIδ-mediated phosphorylation of _82_Ig_83_ constructs unfolded at 57° and CaMKIIδ phosphorylation of unfolded _82_Ig_83_ WT and P-7 mutant after unfolding and S-glutathionylation with 2.0 mM GSH/0.5 mM diamide, detected by autoradiography. Coomassie-stained gel bands are shown above the (γ-P^32^)ATP signal.

Strikingly, in all our heart models, the distal I-band titin region showed a trend toward increased oxidation which became significant when stretch was also included (Figs. 1*E* and 4*A*). This finding is surprising, because the distal Ig domains are considered to be mechanically more stable than the more proximal I-band titin domains (16). Elsewhere, the distal Ig domains were also suggested to be less likely to undergo S-S bonding than the more proximal I-band Ig domains (8). We found that incubation of stretched muscle with GSH and diamide could induce oxidation of more than one cysteine in the same I-band Ig domain (Fig. 2*C*). Based on this information, we were reasonably confident (for additional evidence, see below) that S-glutathionylation but not S-S bonding was the preferential mode of distal Ig-domain oxidation occurring in our in vivo/ex vivo studies. Hence, we focused our subsequent in vitro analyses on S-glutathionylation of distal Ig domains.

A prototypical domain from distal I-band titin is _82_Ig_83_, in which unfolding exposes previously hidden cryptic cysteines and permits them to be S-glutathionylated (6). Interestingly, _82_Ig_83_ was also the domain that showed one of the largest percentages of oxidation change in our preload-increase (shunt) model (123%, cysteine 1; Fig. 1*E*). To test if the _82_Ig_83_ domain is representative of other distal Ig domains with regard to UnDOx, we recombinantly expressed _82_Ig_83_, as well as Ig84, _84_Ig_85_, and a polyprotein consisting of three Ig domains (Ig82-_82_Ig_83_-Ig83), and used different temperatures to thermally unfold the domains in the absence and presence of 2 mM GSH and 0.5 mM diamide. Oxidation was detected by immunoblotting using anti-GSH antibody. UnDOx of all recombinant Ig domains was found to occur when protein unfolding was induced thermally at both 57 °C and 77 °C, but S-glutathionylation was not detected when the proteins remained in their native, folded state at 27 °C (Fig. 4*B*). In addition, S-glutathionylation could be reversed by DTT, as shown for _82_Ig_83_ (Fig. 4 *B*, *Top*). These results prove that UnDOx involving S-glutathionylation can occur in various Ig domains of distal I-band titin in a reversible manner.

Although several distal Ig domains contain two or more cysteines that are available for UnDOx (6–8), our MS data showed that the cysteines in a given domain were not equally responsive to oxidant stress (Figs. 1*E*, 2*C*, and 4*A*). To investigate this further, mutant constructs were made of _82_Ig_83_, which also contains two cysteines, C13585 and C13601 (Fig. 4*C*). The constructs expressed consisted of the WT _82_Ig_83_ containing the two cysteines (CC), a double mutant with both cysteines replaced by alanines (AA), and two single mutants with the substitution of only one of the two cysteines (CA and AC). In WT CC, S-glutathionylation was detected at both 57 °C and 77 °C under oxidative conditions set by incubation with 2 mM GSH and 0.5 mM diamide, while the double mutant AA showed no S-glutathionylation, regardless of temperature. When only the first cysteine (C13585; CA) was present, S-glutathionylation at 57 °C was detected. In contrast, the second cysteine C13601, in the mutant AC, showed no S-glutathionylation under any states of unfolding (Fig. 4*C*). This result was also consistent with the MS data for _82_Ig_83_ oxidation (Fig. 1*E*), suggesting that the first cysteine C13585 is much more reactive than the second cysteine C13601 in _82_Ig_83_. These data also support our reasoning that it is S-glutathionylation (at C13585) rather than intramolecular disulfide bonding (involving both cysteines) that is the preferential type of oxidative modification in this Ig domain.

### S-Glutathionylation Occurs in Sarcomeric I-Bands of Ischemic Mouse Hearts.

To test whether S-glutathionylation may be present within I-band titin under oxidative stress in vivo, we performed GSH immunostaining of mouse heart tissue during ischemia (3 d) induced by ligation of the left anterior descending coronary artery, which is well known to raise intracellular oxidant stress. Ischemic LV samples were compared with nonischemic (remote) tissue from the same heart. Some GSH appeared in a punctuate pattern, likely reflecting its abundance in mitochondria; however, most GSH preferentially localized to the sarcomeric I-bands in ischemic tissue but less so in remote areas, as seen by costaining against I-band titin (Fig. 4*D*). The GSH-fluorescence intensity within I-bands was significantly higher in ischemic vs. remote regions, despite similar SLs (Fig. 4*D*), suggesting that S-glutathionylation of I-band titin is promoted by oxidative stress in vivo.

### UnDOx Triggers Ig-Domain Phosphorylation.

S-glutathionylation prevents the refolding of Ig domains (6) and therefore, potentially exposes other sites for additional modifications, such as phosphorylation. Ca^2+^/calmodulin-dependent protein kinase-II delta (CaMKIIδ) has previously been shown to be activated by oxidation (19). We speculated that this kinase could work in conjunction with UnDOx to modulate titin stiffness, because it phosphorylates elastic elements in cardiac titin and lowers cardiomyocyte passive tension (20). Interestingly, some CaMKIIδ-dependent phosphosites were previously detected in titin Ig domains (20), which prompted us to study the prototypical distal Ig-domain _82_Ig_83_ for its potential to be phosphorylated by CaMKIIδ. We found that computational algorithms [GPS 3.0 (21); Scansite 3.0 (22)] predicted eight potential CaMKIIδ-phosphorylation motifs in _82_Ig_83_. Therefore, we compared recombinant WT _82_Ig_83_ with eight different mutant constructs that contained no or only one possible phosphorylation site (P-0 to P-8; Fig. 4*E*). First, we tested whether unfolding of the domain was required for phosphorylation to occur. Using 57 °C, we unfolded the domains and then allowed CaMKIIδ-mediated phosphorylation to occur in the presence of radioactively labeled [γ-P^32^] adenosine triphosphate. We found that only the WT and P-7 mutant _82_Ig_83_ were able to be phosphorylated, and only if the domain was first unfolded (Fig. 4*E*). Thus, CaMKIIδ can phosphorylate the site P-7 in the unfolded _82_Ig_83_ domain, which is serine S13610 in the mouse consensus titin sequence (UniProtKB ID A2ASS6). To then evaluate the effect of UnDOx on phosphorylation, WT and P-7 were S-glutathionylated prior to phosphorylation, via incubation with 2 mM GSH and 0.5 mM diamide. Interestingly, both the WT and the P-7 mutant showed enhanced phosphorylation after S-glutathionylation, compared with thermal unfolding alone (Fig. 4*E*). We conclude that phosphorylation of titin Ig domains requires their unfolding, and oxidant stress via S-glutathionylation can further promote the phosphorylation events at specific residues, as shown in a representative distal I-band domain.

### UnDOx Promotes Aggregation, Unfolding, and Nonconstitutive Folding.

**Fig. 5.**
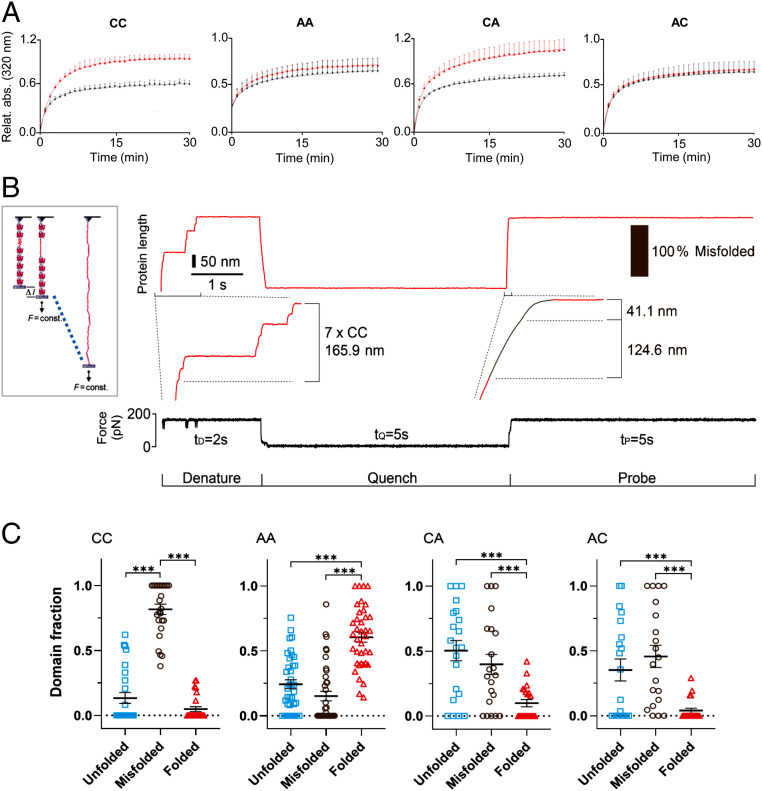
Molecular consequences of UnDOx of _82_Ig_83_. (*A*) Aggregation of unfolded wild-type (CC) and mutant _82_Ig_83_ (AA, CA, and AC) under control (black) and oxidative (2 mM GSH/0.5 mM diamide; red) conditions. Absorbance from partially refolded construct measured at 320 nm and 1-min intervals for 30 min. CC: control *n* = 4, oxi *n* = 5. Double mutant, no cysteines (AA): control *n* = 4, oxi *n* = 4. Single mutant C13601A (CA): control *n* = 6, oxi *n* = 5. Single mutant C13585A (AC): control *n* = 6, oxi *n* = 4. Data are normalized to highest aggregation value of _82_Ig_83_ (CC) after oxidation. Data are means ± SD. Curves are exponential saturation function best fits. (*B*) Unfolding/refolding of recombinant polyprotein (_82_Ig_83_)_8_ measured by single-molecule AFM force spectroscopy (*Left*) and example trace with seven domains in the tether. In presence of 1 mM GSH/2 mM diamide, domains were first unfolded at 175 pN and held extended for 2 s (denature). Then, force was quenched to zero for 5 s to allow for refolding (quench), before it was increased back to 175 pN (probe). (*C*) Proportion of unfolded, misfolded, and folded events detected in S-glutathionylated _82_Ig_83_ CC (*n* = 130), AA (*n* = 209), CA (*n* = 113), and AC (*n* = 98) polyproteins. Data are mean ± SEM; ***P < 0.001, by ANOVA followed by Tukey’s multiple comparisons test.

We considered it likely that UnDOx also impacts intra- and intermolecular domain interactions. To gain an understanding of what effect UnDOx may have on the propensity for nonnative interactions among titin Ig domains, we carried out aggregation tests with recombinant _82_Ig_83_. Many proteins are unable to independently regain their physiological conformation when diluted from a denaturing environment into a physiological buffer, with a common reaction being the aggregation of these “partially unfolded” proteins. Since _82_Ig_83_ showed differential oxidation of the two cysteines, we wanted to know if UnDOx of the specific cysteines could differentially affect domain stability. Aggregation of recombinant WT (CC) and mutant _82_Ig_83_ (AA, CA, and AC) measured by light scattering was detected for all constructs, with a rapid increase in aggregation detected in the first few minutes, until a plateau was reached after ∼25 min (Fig. 5*A*). Interestingly, UnDOx induced by incubation with 2 mM GSH and 0.5 mM diamide increased aggregation by ∼35% in the WT CC and mutant construct CA. In contrast, the mutant constructs AC and AA showed no additional increase in aggregation. This demonstrated that S-glutathionylation of the first cysteine (C13585) within _82_Ig_83_ promoted aggregation of the domains, likely by stabilizing the unfolded state.

Next, we directly tested if the additional aggregation was related to the reduced ability for _82_Ig_83_ to properly refold under oxidizing conditions. To this end, we used a homopolyprotein mutant construct containing eight identical _82_Ig_83_ domains. The construct was investigated by single-molecule force spectroscopy using a specialized atomic force microscope (AFM) that allowed the stretching of the constructs under a constant force (Fig. 5 *B*, *Inset*). The domains were exposed to 2 mM GSH and 0.5 mM diamide and then mechanically unfolded at a force of 175 pN. This produced a staircase-like trace of length changes (each step, 23.7 nm), which are fingerprints of the consecutive unfolding events of the domains (Fig. 5*B*). The force was then quenched and returned to zero for 5 s to allow refolding to occur, followed by a probe pulse of again 175 pN. By comparing the number and size of the steps in the denaturing and probe pulses (6), we could determine whether the domains remained unfolded after the quench (no or reduced number of regular steps in the probe pulse), misfolded (irregular step sizes in the probe pulse), or had correctly folded into the native state (same step sizes in both the denature and probe pulses).

Under oxidant stress, the WT _82_Ig_83_ domain was found to have a high rate of nonconstitutive folding (“misfolding,” 81 ± 4%; CC; Fig. 5*C*). The double mutant (AA) revealed a relatively low frequency of misfolding and unfolding events (15 ± 4% and 24 ± 3%, respectively) and was able to successfully refold 61 ± 4% of the time during the probe pulse. When either one of the two cysteines was present, the proportion of misfolding was similar (40 ± 8% for CA and 46 ± 8% for AC); however, the percentage of unfolded domains detected was higher in CA (50 ± 8%) than in AC (35 ± 8%; Fig. 5*C*). The high frequency of misfolded and unfolded domains under oxidizing conditions when the first or both cysteines are present, suggests that the S-glutathionylation (of only one cysteine in _82_Ig_83_) stabilizes the unfolded state of the Ig domain and increases nonnative intramolecular interactions. In summary, UnDOx involving S-glutathionylation both hinders constitutive folding (Fig. 5*C*) and promotes aggregation (Fig. 5*A*) of a prototypical distal I-band titin Ig domain.

## Discussion

One of the most important functions of titin is the mediation of passive stiffness in striated muscle (23). A mechanism to modulate passive stiffness is UnDOx, whereby the stretching of sarcomeres causes the unfolding of titin Ig domains to expose cryptic cysteines that can then undergo oxidative modifications (6, 10). These modifications have been suggested to alter the passive stiffness of titin in vitro (6–8, 24) but have not specifically been demonstrated in vivo. We now show that elastic titin from heart and skeletal muscle becomes preferentially oxidized ex vivo or in vivo under oxidizing conditions in the presence of increased strain. The oxidation changes we have detected only represent sites capable of reversible oxidation due to the reduction step required for the differential ICAT labeling. Therefore, we argue that the oxidative modifications of these titin regions are unlikely to be an indicator of oxidative damage of the cardiac and skeletal muscle cells, but instead may play a strong regulatory role in the dynamic adjustment of titin’s molecular properties and mechanical function. Unexpectedly, the main titin region undergoing reversible oxidative changes in the heart is the distal I-band Ig-domain segment. The functional consequences of UnDOx within the distal titin spring include oxidation type-dependent effects on cardiomyocyte passive tension, domain (mis)folding, phosphorylation, and homotypic titin interactions serving elastic filament stabilization.

By using mechanical experiments on isolated human cardiomyocytes, we confirmed and extended the finding (6) that UnDOx involving S-glutathionylation causes a decrease in titin-based stiffness. This mechanism has also recently been shown to be relevant to skeletal muscle (25), suggesting that UnDOx through S-glutathionylation is an important regulatory mechanism, to varying degrees, in all striated muscle types. However, it has also been speculated that disulfide-bond formation within titin Ig domains might cause an increase in titin-based stiffness due to the intradomain cross-linking (6). Further, it has been shown that S-S bonding is triggered by the oxidized form of PDI, which, among other effects, increases the rate of refolding of the unfolded titin Ig domains (11). We have now demonstrated that oxidized PDI causes an increase in cardiomyocyte passive stiffness when the titin domains first become unfolded by stretching. These results would appear to demonstrate contradictory modes of UnDOx; however, the stabilization of the unfolded domains through S-glutathionylation, followed by PDI-mediated disulfide bonding of multiple Ig domains simultaneously, may enable the cooperative fine tuning of cardiac stiffness during oxidative stress on nearly a beat-by-beat basis.

UnDOx also opens titin domains up for additional modifications such as phosphorylation. The mechanical effect of phosphorylation of titin has so far been studied exclusively for the unstructured elements (N2Bus and PEVK) and the N2A region within the I-band spring (17). We could show that a phosphorylation site within Ig domain _82_Ig_83_ (S13610 in mouse titin) is available after unfolding and that UnDOx can enhance phosphorylation by CaMKIIδ at this site. The enhanced phosphorylation is very likely due to the S-glutathionylation of _82_Ig_83_ maintaining the unfolded state of the domain, facilitating the subsequent phosphorylation with CaMKIIδ. Another possibility is that S-glutathionylation of _82_Ig_83_ enhances the affinity of CaMKIIδ for _82_Ig_83_. An increase in the activity of CaMKIIδ by oxidative stress (19) can be ruled out, since the oxidants were inactivated by *N*-ethylmaleimide before phosphorylation. Regardless of the exact mechanism of the increased phosphorylation after oxidation of _82_Ig_83_, important insight into a relationship between S-glutathionylation and phosphorylation of titin has been obtained. We propose that this interaction is an additional “tool” to modulate titin-based passive stiffness.

Closer investigation into which titin spring regions show UnDOx in vivo revealed that many distal Ig domains were consistently oxidized in all our heart and skeletal muscle models. Moreover, the combination of strain and oxidative stress in the shunt model caused preferential oxidation of distal vs. proximal I-band titin domains. We confirmed that _82_Ig_83_, Ig84, and _84_Ig_85_, all located within the distal I-band, could undergo UnDOx involving S-glutathionylation in vitro. Interestingly, the frequently oxidized cysteines within these domains (C13585 [_82_Ig_83_], C13828 [Ig84], and C13941 [_84_Ig_85_]) have a microenvironment similar to that of a potential S-glutathionylation site (26). Each of the cysteines are flanked by negatively charged residues (E or D), and further afield, after 6 to 10 amino acids, are positive lysines (*SI Appendix*, Table S3). In contrast, the cysteines which were less strongly oxidized (e.g., C13601 [_82_Ig_83_]) have no such sequence motif. These considerations support our interpretation of the MS results as being highly suggestive of S-glutathionylation as the most likely type of oxidation in the distal I-band Ig domains of our heart and muscle models. Furthermore, increased S-glutathionylation was clearly confirmed to occur in the sarcomeric I-band regions of ischemic mouse cardiomyocytes in vivo, substantiating our in vitro findings.

The consistent oxidation increase in the distal Ig domains was unexpected, because single-molecule force spectroscopy measurements on recombinant titin Ig domains have previously demonstrated that these domains generally unfold at higher stretch forces than the proximal Ig domains (10, 16). The relatively high mechanical stability of the distal Ig domains does not mean that they never unfold in situ, but unfolding events might be less frequent than in the proximal domains. Notably, oxidation of distal Ig domains was still detected in our “low-stretch” heart models (Langendorff perfusion and afterload increase), even though domain unfolding was clearly essential for the S-glutathionylation to occur in vitro. We thus speculate that a few distal Ig domains do not adopt a fully folded conformation under physiological conditions (even when strain is low), but rather maintain an S-glutathionylation-controlled, partially unfolded state allowing “regulated aggregation.” Titin aggregation has previously been observed under in vitro conditions in the N2A region and in other Ig domains following their unfolding (27–29), and we have been able to directly demonstrate it for _82_Ig_83_, with enhanced aggregation seen after S-glutathionylation-associated UnDOx. Further, while we are confident that S-glutathionylation is the dominant type of distal Ig-domain oxidation in our in vivo models, we cannot completely rule out the existence of S-S bonded, folded, or unfolded domains. However, even S-S-bonded Ig domains of titin are predicted to have an increased propensity for aggregation, as they would unfold at lower forces and have reduced mechanical stability compared to reduced domains (8, 30), and therefore be more likely to misfold.

**Fig. 6.**
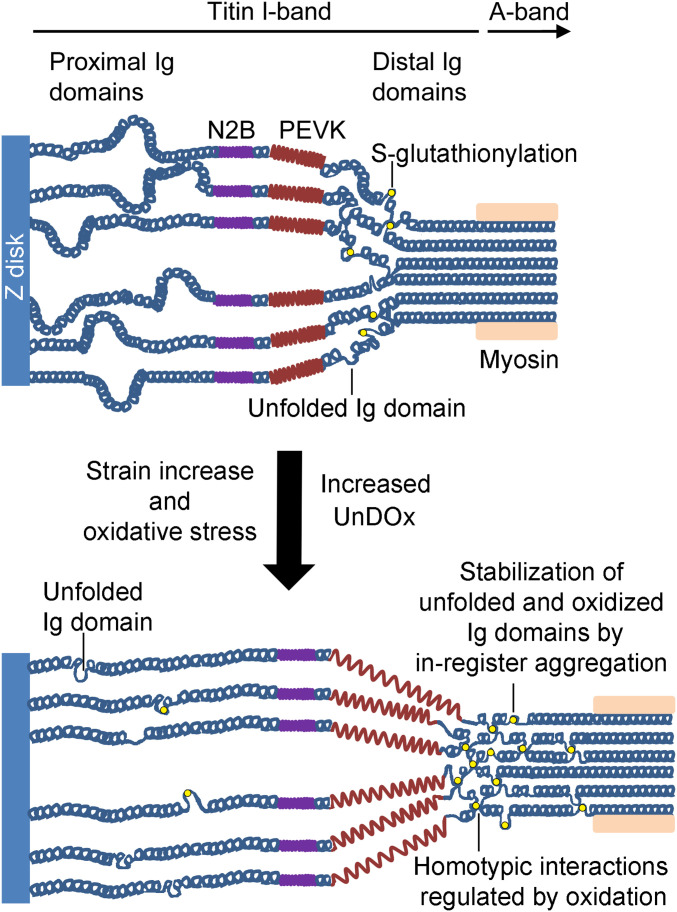
Proposed mechanism of UnDOx to control in-register aggregation of distal I-band titin. Several Ig domains from the distal I-band titin region (mainly the NH_2_-terminal half) are suggested to be in a partially unfolded state in vivo. S-glutathionylation of the unfolded domains stabilizes the unfolded state, which promotes aggregation of parallel titin molecules. Stretch under oxidant stress conditions recruits more Ig domains to the unfolded state and further promotes this mechanism. The result is an improved anchorage of the titin spring under mechanical and oxidant stress.

Aggregation is often thought of as a negative consequence of protein unfolding and is associated with failing proteostasis and chaperone regulation (31). However, this is unlikely to be the case for the distal I-band titin region, because chaperones have not been found to bind to titin’s distal Ig domains in vivo, whereas they do associate under stress with the proximal and middle Ig-domain segments and the N2A region of titin, protecting them from aggregation (29, 32). The apparent lack of chaperone protection for the distal titin Ig domains, combined with our findings of consistent oxidation of distal Ig domains, leads us to suggest that the observed UnDOx-associated aggregation is important for the physiological suprastructure and function of the distal spring segment. According to this scenario, distal I-band titin is stabilized by the in-register aggregation of titin molecules, which aids in the synchronization and propagation of tension changes. The distal I-band titin region has long been thought to consist of composite filaments made up of in-register aggregates of six titin molecules, which emerge from each tip of the thick (myosin) filament (33). We propose that these so-called “end filaments” are regulated in their homotypic interactions by oxidative modifications (Fig. 6). Beyond the end filament in the direction of the Z-disk, where the PEVK element adjoins, the composite titin filament presumably separates into individual strands, which may be needed for an optimal elastic function (29). UnDOx-controlled titin aggregation within the end-filament region then allows to dynamically adjust the COOH-terminal anchorage of the titin spring, depending on the level of strain and oxidative stress. Additional modifications of disulfide bonding and phosphorylation could aid in the overall tuning of titin anchorage and passive tension. If these changes persisted for longer periods, such as under conditions of chronic ischemic heart disease, titin and associated sarcomere proteins would probably be turned over and replaced (34). However, short-term oxidative modifications within the distal titin spring are very likely to be reversible under physiological conditions, such as exercise and fatigue (35), thus permitting control over the properties of the end filament through malleable intertitin interaction.

Our study paves the way for interventions that target the stiffness of titin in common forms of heart failure, particularly heart failure with preserved ejection fraction (HFpEF) (4, 17). This disease is characterized by pathological myocardial stiffening, presumably as a consequence of metabolic risk triggering microvascular endothelial inflammation and oxidative stress (17). The normalization of titin-based stiffness in HFpEF has previously been attempted in animal models by improving titin phosphorylation (4, 17). However, our work implicates oxidative modifications of titin as an attractive, alternative target for treatment. A limitation of the present study is that we have not distinguished between S-glutathionylation and S-S bonding of titin in our ex vivo and in vivo heart models. This distinction is important, because increased S-glutathionylation of I-band titin both stabilizes the distal titin springs and lowers cardiomyocyte stiffness, whereas increased S-S bonding within the N2Bus region (5) and the more proximal Ig domains (8) of elastic titin elevates cardiomyocyte stiffness. Both types of modification could be explored with regard to therapeutic interventions. As a prerequisite before testing such a potential treatment strategy, HFpEF heart tissues from humans and animal models should be analyzed for oxidative titin modifications, and the type of modification be determined. Finally, oxidative modifications of titin could be targeted jointly with titin phosphorylation deficits in HFpEF, to more effectively improve cardiac compliance and ventricular filling in patients.

## Materials and Methods

For details, see *SI Appendix*.

### Mouse Models Studied for Peptide Oxidation.

Male C57BL/6N mice (Charles River Laboratories) were used at the age of 5 wk, and the heart and skeletal (leg) muscles prepared. All procedures conformed to the institutional guidelines, following the animal care and use protocols. The following models were prepared: 1) Langendorff-perfused hearts under control conditions (*n* = 3) or perfused with buffer containing 0.1% H_2_O_2_ (*n* = 3) or 1 mM DTT (*n* = 3). 2) Afterload-increase model in working-heart mode (13) studied under control conditions (80 mmHg for 15 min; *n* = 4) or after pressure increase to 120 mmHg (15 min; *n* = 4). 3) Preload-increase in vivo model (14, 15) produced by inducing an aortocaval shunt (*n* = 4) and sham-operated mice as controls (*n* = 3). 4) Stretched or nonstretched skeletal muscles (vastus lateralis) exposed to solution containing 2 mM GSH and 0.5 mM diamide (stretch oxidized, *n* = 3; nonstretch oxidized, *n* = 3); nonstrained, nonoxidized muscles were used as controls (nonstretch CTRL; *n* = 3).

### Oxidation Detection (OxICAT).

The cysteine redox state was determined by OxICAT (12). This method uses the cysteine-specific ICAT reagent (Applied Biosystems). Reduced cysteines are specifically, differentially labeled with the ^12^C-ICAT and oxidized cysteines after their reduction with the ^13^C-ICAT. The ICAT reagent also contains an additional affinity tag allowing tagged peptides to be isolated and concentrated before MS. OxICAT labeling was performed as previously described (36).

### Mass Spectrometry.

ICAT-labeled peptides were analyzed by reverse-phase nano-liquid chromatography (LC) coupled to an LTQ Orbitrap Elite MS/MS instrument (Thermo Fisher Scientific) (37). Raw data obtained by LC-MS were evaluated with MaxQuant (v1.3.0.5) and peptide sequences from fragmentation spectra identified by Andromeda search (38) against the mouse proteome (UniProtKB ID UP000000589). ICAT-0 (^12^C-form) was chosen for light labeling and ICAT-9 (^13^C-form) for heavy labeling. ICAT pairs in the MS spectrum were evaluated using Mass++ v2.7.4. Spectra from frequently detected Ig domains are shown in *SI Appendix*, Fig. S2. The percentage of oxidation change comparing the different model groups was calculated for the individual peptides for each sample using Perseus software (v1.5.5.3) and displayed on volcano plots. Data are available via ProteomeXchange (https://www.ebi.ac.uk/pride/) with identifier PXD018174.

### Myocardial Ischemia Model.

Permanent myocardial infarction was produced in C57BL/6N mice by suture occlusion and maintained for 3 d (34). Cardiac tissue was separated into ischemic LV tissue and nonischemic (remote) tissue. Procedures were conducted with the approval of the authorities (Landesamt für Natur, Umwelt und Verbraucherschutz Nordrhein-Westfalen; reference number 84-02.04.2013.A122).

### GSSG:GSH Ratio Determination.

The content of GSSG and the total glutathione concentration (GSH+GSSG) of cardiac and skeletal muscle lysates was measured as described (13).

### Recombinant Protein Expression, Purification, and Mutagenesis.

The cDNAs coding for titin Ig domains were cloned into pGEX-4T1 vectors and transformed into competent *Escherichia coli* (XL1 blue). Proteins were expressed in *E. coli* BL21(DE3) and purified (6). Amino acid sequences of WT constructs are shown in *SI Appendix*, Table S3. Mutant _82_Ig_83_ constructs were generated using the QuikChange site-directed mutagenesis kit (Stratagene); sequences are shown in *SI Appendix*, Table S4. PDI A1 was expressed and purified as described (11) and treated with 0.03% H_2_O_2_ to obtain the oxidized form.

### S-Glutathionylation and Phosphorylation of Ig Domains.

Recombinant Ig-domain proteins were incubated with 2 mM GSH and 0.5 mM diamide for 30 min at 27 °C, 57 °C, or 77 °C in a shaker; 5 mM DTT was also used to reverse oxidation. Proteins were analyzed by immunoblotting using anti-GSH antibody (ab19534, Abcam; 1:2,000). Phosphorylation by CaMKIIδ (Merck Millipore) was detected by autoradiography.

### Immunofluorescence Staining of Myocardial Tissue.

Ischemic and remote mouse myocardial tissue was immunostained against GSH and I-band titin, as described (32).

### Photometric Detection of Aggregation.

Recombinant WT and mutant _82_Ig_83_ constructs were unfolded in 6 M guanidine hydrochloride solution and partially back folded in 30 mM Tris/HCl, 50 mM KCl, just before measuring absorbance at 320 nm every 2 s for 30 min (29).

### Mechanical Measurements on Skinned Cardiomyocytes.

Human LV tissue (nontransplanted donor heart) was obtained from the Sydney Heart Bank (39). Sample collection was done in full accordance with Australian National Health Medical Research guidelines and approved by the Human Research Ethics Committee of the University of Sydney (2012/2814). Informed consent was obtained from the next of kin of the donors. Skinned cardiomyocytes were prepared from deep-frozen heart tissue and mechanical measurements performed, as described (6).

### AFM Single-Molecule Force Spectroscopy.

Force spectroscopy experiments were conducted in force-clamp mode, using a commercial AFM (Luigs and Neumann) (40). Preparation and purification of the 8-Ig domain _82_Ig_83_ polyprotein were as described (6).

### Statistical Analysis.

Statistical analysis was performed in GraphPad Prism v6. Significance was determined using unpaired, two-tailed Student’s *t* test for parametric tests or Mann–Whitney test for nonparametric tests with significance considered at *P* < 0.05. Comparisons of more than two groups were first done using ANOVA, followed by Tukey’s multiple comparisons test.

## Supplementary Material

Supplementary File

## Data Availability

The ICAT LC-MS data have been uploaded to the EBI PRIDE consortium repository accessible at https://www.ebi.ac.uk/pride/ (41) with the dataset identifier PXD018174 (42). Materials will be made available upon reasonable request.
