# Supplementary material for: Regulation of titin-based cardiac stiffness by unfolded domain oxidation (UnDOx)
Source: Proc Natl Acad Sci U S A. 2020 Sep 14;117(39):24545–56. doi: 10.1073/pnas.2004900117 (PMC7533878; doi:10.1073/pnas.2004900117)
Supplement: Supplementary File [file pnas.2004900117.sapp.pdf]

Supplementary Materials, Loescher et al.

Supplementary Methods

Mouse heart and skeletal muscle models

Heart and skeletal muscle models were generated from male C57BL/6N mice (Charles River, Wilmington, USA; age, 5 weeks). Animals were anesthetized with isoflurane 100% (v/v), rimadyl 4 mg/kg body weight and 250 I.U. heparin sodium anticoagulated. They were killed by cervical dislocation and the heart and skeletal (leg) muscles were prepared. All procedures conformed to the institutional guidelines, following the animal care and use protocols. The following models were used to measure changes in the oxidation state via OxICAT and mass spectrometry:

1.) *Ex-vivo* hearts perfused according to the Langendorff method for 20 min with either Krebs-Henseleit (KH) solution (mM: 137 NaCl, 2.7 KCl, 5 $MgCl_2$, 2 EGTA, 10 Tris, pH 7.4) for control conditions (N=3) or with KH solution containing 0.1 % (v/v) H_2O_2 (oxidizing; N=3) or 1 mM DTT (reducing; N=3).

2.) *Ex-vivo* hearts in ‘working heart’ mode, prepared as described (*1*). The heart was removed and the aorta and pulmonary vein were cannulated. Warmed Tyrode’s solution (mM: 118.5 NaCl, 25 $NaHCO_3$, 4.7 KCl, 1.2 $MgSO_4$, 1.2 KH_2PO_4, 2.5 $CaCl_2$, 0.5 EDTA, 11.1 glucose, pH 7.4, 37°C) was injected through the pulmonary vein at a set preload (10 mmHg). Through the active contraction of the heart, the Tyrode's solution was pumped through the aortic cannula which was connected to a post-load column, allowing post-load pressure to be increased with a post-load resistance membrane (80 mmHg for control). The hearts were perfused in working-heart mode under control conditions at 80 mmHg for 15 min (N=4). For the increase in afterload, pressure was increased to 120 mmHg and the heart was perfused for an additional 15 min (N=4).

3.) The aortocaval shunt was produced in mouse hearts through a vascular connection between the abdominal aorta and the inferior vena cava (N=4); this procedure generates increased preload *in vivo*. We previously showed that this model has greatly enlarged left ventricles and generates intrinsic oxidative stress (*2, 3*). The procedure was conducted on 8-week-old mice and the mice were sacrificed at 12 weeks of age. Sham-operated mice were used as controls (N=3). Our previous analyses showed the same titin isoform composition in shunt and sham hearts (*3*).

4.) *Ex-vivo*, stretched or non-stretched, skeletal muscle preparations (vastus lateralis and vastus medialis). For the stretched muscle model, the back legs were severed just below the groin and the upper skin layer removed. The vastus lateralis and vastus medialis were stretched by connecting the foot to the thigh. The degree of stretch (~20%) was determined by measuring the shortened length between the foot and thigh. The epimysium was then opened and the mouse legs were incubated in KH solution for 20 min at room temperature. For oxidative conditions, muscles were exposed to 2 mM GSH and 0.5 mM diamide in KH solution (Stretch-oxidized, N=3; Nonstretch-oxidized, N=3). Diamide is permeable to cellular membranes and induces oxidative stress by converting native GSH into GSSG. Diamide was used in combination with GSH to prevent the cellular depletion of GSH. Although GSH itself is not cell permeable, there is evidence that when applied together with diamide, it can enter the cell through aqueous pores induced by diamide (*4*). In addition, non-strained, non-oxidized legs were used as controls (Nonstretch-CTRL; N=3). MS and GSSG:GSH ratio analysis were performed using *m. vastus lateralis*.

www.pnas.org/cgi/doi/10.1073/pnas.2004900117

Myocardial ischemia mouse model

For induction of permanent myocardial ischemia (*5*), C57/BL6J mice were anesthetized by intra-peritoneal injection of ketamine (60 mg/kg body weight (BW)) and xylazine (10 mg/kg BW) and ventilated with a tidal volume of 200 µl at a rate of 140 strokes/min, with a mixture of two thirds air, one third oxygen and isoflurane 2.0-2.5 Vol.% (Forene ®, Abbott GmbH, Germany). Mice were placed in a supine position on a warmed plate. Body temperature was maintained at 37°C, and electrocardiography (ECG; Hugo Sachs Elektronik—Harvard Apparatus, March, Germany) was monitored. After left lateral thoracotomy between the third and fourth rib, the pericardium was dissected and a 7-0 surgical suture was cautiously passed underneath the left anterior descending coronary artery at a position 1 mm from the tip of the left auricle. Myocardial ischemia was produced by suture occlusion. The correct position of the suture was confirmed by blanching of the apex and characteristic change in echocardiography (ST-elevation). Permanent myocardial infarction was performed for 3 days. All animals received buprenorphine (0.05 – 0.1 mg/kg BW, s.c.) every 8 h. Mice were sacrificed by cervical dislocation and hearts were excised. Cardiac tissue was separated into ischemic left ventricular tissue and non-ischemic (remote) tissue (cardiac tissue above the occlusion and interventricular septum) and stored at -80°C.

Oxidation detection (OxICAT)

In the OxICAT method (*6*), reduced cysteines are specifically, differentially labeled with the ^{12}C-ICAT and oxidized cysteines after their reduction with the ^{13}C-ICAT (Applied Biosystems, Waltham, MA, USA). The ICAT reagent also contains an additional affinity tag allowing tagged peptides to be isolated and concentrated before MS analysis. OxICAT labeling was performed as described (*7*). Briefly, ~100 µg of heart (left ventricular) or skeletal muscle tissue was mixed with anaerobic denaturing alkylation buffer (mM: 6000 urea, 10 EDTA, 200 Tris HCl pH 8.5, 0.5% SDS (w/v) and 20% acetonitrile (ACN), and homogenized at 1500 rpm. The homogenate was mixed with the light ICAT and incubated for 2 h at 37°C and 1300 rpm, protected from light in a shaker. Then 500 µL precooled (-20°C) acetone was added to the reaction mixture and the proteins were precipitated overnight at -20°C.

Precipitated proteins were sedimented by centrifugation (30 min, 4°C, 16’000 x g) and the resulting pellet washed twice in 500 µL precooled acetone to remove excess light labeling reagent. The acetone was removed and residual acetone was evaporated off for ~5 min. The protein pellet was then resuspended in denaturing alkylation buffer. Non-labeled oxidized cysteines were reduced using 50 mM TCEP and incubated for 10 min at 300 rpm and 37°C in a shaker. After reduction, the heavy ICAT was spiked with 20 µL ACN and added to the reaction mixture. Newly reduced thiols were alkylated for 2 h at 37°C and 1300 rpm in a shaker. The reaction was again quenched with acetone as above and proteins precipitated.

The precipitated proteins were again sedimented, the pellet was washed twice with ice cold acetone and the resulting pellet was digested overnight at 37°C in a mixture of 80 µL denaturing buffer (50 mM Tris, 0.1% SDS (w/v), pH 8.5), 20 µL ACN and 100 µL of a 0.125 µg/µL trypsin solution. Tagged peptides were then isolated and concentrated using cation exchange and affinity chromatography (AB SCIEX according to the manufacturers protocol with “Affinity Buffer-Elute” (30% ACN, 0.1% TFA (trifluoroacetic acid)) freshly prepared at the day of the experiment). The isolated ICAT-labeled peptides were evaporated in the vacuum centrifuge for about 8 hours, to complete dryness. The resulting pellet was resuspended in a 95:5 mixture of cleavage reagent A and cleavage reagent B (AB SCIEX) to remove the biotin tag. The mixture was then incubated for 2 h at 37°C and 300 rpm in a shaker. The cleavage reagent was evaporated in the vacuum centrifuge for about 20 min. After evaporation, the pellet was resuspended with 0.1%

trifluoroacetic acid to a final peptide concentration of 200 ng per 15 µL. Samples were stored at -80°C until MS analysis.

Mass spectrometry data analysis

ICAT-labeled peptides were analyzed by reverse phase nano-LC coupled to a high-resolution mass spectrometer (LTQ Orbitrap Elite, Thermo Fisher Scientific, Waltham, USA), as described (*8*). Raw data obtained by LC-MS/MS analysis were evaluated with the program MaxQuant (version 1.3.0.5) (*9*). To identify the peptide sequences from the corresponding fragmentation spectra, the search algorithm Andromeda (integrated into MaxQuant) was used (*9*). The search was performed against the mouse proteome from the UniProt database (UniProt-ID, UP000000589), with a false discovery rate of 1% at peptide level. The quant module was used for the detection of the isotope patterns and the ICAT pairs of the individual peptides, using the following parameters: trypsin selected as endopeptidase with a maximum of two permissible false cleavages; and oxidation of methionine and acetylation of the N-terminus of proteins considered as variable modifications. ICAT-0 (ICAT in the ^{12}C-form) was chosen for the light labeling and ICAT-9 (ICAT in the ^{13}C-form) for the heavy labeling. To visually evaluate ICAT pairs in the MS spectrum, the program Mass++ 2.7.4 was used. Spectra from frequently detected Ig-domains are displayed in Fig. S2.

The percentage oxidation change was calculated for the individual peptides for each sample. The heavy ICAT signal was divided by the total signal (heavy signal + light signal) for that peptide to determine the percentage of the signal due to oxidation for each peptide:

$$\frac{\text{Heavy (H) signal}}{\text{Total signal}} = \text{Pecentage of signal from oxidation}\,.$$

The average percentage oxidation was determined for each peptide for a given experimental group. The data was then further processed using the Perseus software v1.5.5.3 *(10)* for the removal of contaminants, log2 transformation of the percent oxidation for each peptide, median subtraction for data normalization and p-value calculations, and data display on volcano plots.

In the volcano plots, determined using the Perseus software *(10)*, the -log10 of the p-values (Y-axis; p>1.3 or p<-1.3 considered significant) was plotted against the difference between the oxidant stress group and control (X-axis; between -0.58 and 0.58 considered unchanged):

$$\log 2\ (\%\ \text{Oxidation stress}) - \log 2\ (\%\ \text{Oxidation control}) = \log 2\ \left(\frac{\%\ \text{Oxidation stress}}{\%\ \text{Oxidation control}}\right).$$

The titin peptides were assigned to the associated titin domains and the exact position of the cysteines was determined using the canonical titin sequence of the mouse from the UniProt database (entry, A2ASS6).

The transformation of the $\log 2\ (\frac{\%\ \text{Oxidation stress}}{\%\ \text{Oxidation control}})$ into relative percentage oxidation change was used for the colored bar graph comparisons shown in Figures 1 and 2 (comparison of I-band vs. A-band and proximal vs. distal I-band titin oxidation changes). An increase in oxidation by >50% (1.5-fold increase; this is log2 % oxidation change >0.58) was displayed as a significant change.

The MS proteomics data have been deposited to the ProteomeXchange Consortium via the PRIDE (*11*) partner repository (https://www.ebi.ac.uk/pride/) with the dataset identifier PXD018174.

GSSG:GSH ratio determination

The content of GSSG and the total glutathione concentration (GSH and GSSG) were measured, as follows (*1*). Lysates were made from either 50 µg protein in GSH buffer (mM: 106.1 K_2HPO_4;

18.7 KH_2PO_4; 6.3 EDTA, pH7.4) alone for the total glutathione determination or in 250-500 µg protein GSH buffer containing 150 mM 1-methyl-2-vinylpyridine trifluoromethane sulfonate (for GSSG determination) and then homogenized at 1500 rpm. Lysates were then transferred into 1 mL reaction buffer (mM in GSH buffer: 0.6 sulfhydryl reagent 5,5′-dithio-bis(2-nitrobenzoic acid) (DTNB); 0.4 6 5'5'dithiobis-2-nitrobenzoic acid (NADPH)). Glutathione reductase (451 units/mL) was added just before the measurement and absorbance at 412 nm was measured for 10 min at 30 s interval in a photometer. From the measured absorption curve, the slope was determined and used to calculate the concentration of GSSG or total glutathione. Reduced GSH content was calculated as the difference between total glutathione minus the values obtained for GSSG.

Recombinant protein expression, purification, and mutagenesis

For the production of the specific Ig domains, specific 3' and 5' PCR primers were obtained from Eurofins Scientific (Luxemburg, LU) which also contained terminal sequences for restriction enzymes; a human cDNA library was used for the amplification. The cDNAs coding for titin Ig domains were cloned into pGEX-4T1 vectors, which also contained a GST tag to generate a fusion protein, and transformed into heat shock competent *E.coli* (XL1 blue). Sequencing of the inserted plasmid vector was performed and the program Geneious 5.6.7 was used for the alignment with the respective reference sequences to confirm the correct sequence. Proteins were expressed, as previously described (*12*), in *E.coli* BL21(DE3) and vector expression was induced with IPTG. Proteins were purified after cell lysis using glutathione sepharose matrix as per the manufactures instructions (GE Healthcare, Little Chalfont, GB). Amino acid sequences of wildtype Ig domain constructs expressed are shown in Table S1. $_{82}$Ig$_{83}$ mutants were generated using the QuikChange® site-directed mutagenesis kit (Stratagene).

For the production of the $_{82}$Ig$_{83}$ cysteine mutants, the wt $_{82}$Ig$_{83}$ fragment was mutated using the QuikChange® site-directed mutagenesis kit (Stratagene), according to standard protocols. For the phosphosite mutants, $_{82}$Ig$_{83}$ containing none of the predicted phosphorylation sites (P-0) was obtained from Biomers (Ulm, Germany) and used as the starting sequence for the QuikChange® kit. All fragments were sub-cloned into vector pGEX-4T1, validated by sequencing and expressed as described above. Amino acid sequences of mutant constructs of $_{82}$Ig$_{83}$ are shown in Table S2.

The peT 15b plasmid containing the PDI A1 sequence was kindly supplied by Dr. Julio Fernandez’s lab and was also expressed and purified as previously described (*13*). To make the oxidized form of PDI, the expressed peptide was treated with 0.03% hydrogen peroxide overnight and then washed with the appropriate experimental buffer.

S-glutathionylation and phosphorylation of Ig-domains

For S-glutathionylation of recombinant Ig-domains, proteins were incubated with a mixture of 2 mM GSH and 0.5 mM diamide for 30 min at 27°C, 57°C or 77°C on a shaker. To reverse oxidation, 5 mM DTT were applied. After incubation, 5 µg of protein (prepared without any reducing agent) were used for SDS-PAGE and western blotting using mouse monoclonal anti-glutathione antibody ([D8]; ab19534, Abcam; 1:2,000), followed by secondary antibody, anti-mouse IgG (H+L) HRP Goat (115-035-146, Dianova; 1:10,000). Documentation was done with a luminescence image analyzer (LAS 4000; GE Healthcare). To determine the interaction between S-glutathionylation and phosphorylation, GSH and diamide were inactivated by addition of 75 mM N-ethylmaleimide prior to phosphorylation. *In-vitro* phosphorylation of the recombinant Ig-domains was catalyzed by CaMKIIδ (Merck Millipore, Billerica, USA) in presence of calmodulin (Abcam, Cambridge, UK) using the following buffer: 200 mM $CaCl_2$, 100 mM $MgCl_2$, 20 mM N-ethylmaleimide, 1 mM EDTA, 500 mM Tris pH 7.5, 0.1% Brij 35 (w/v). Phosphorylation was

detected by autoradiography using radioactive [γ-P^{32}] ATP (SRP-301; 250 µCi/µM; Hartmann Analytic, Braunschweig, Germany) in presence of 2 mM ATP.

Mechanical measurements on skinned cardiomyocytes

Skinned human cardiomyocytes were prepared from deep-frozen donor heart tissue obtained from the Sydney Heart Bank (*14*) and mechanical measurements performed, as previously described (*12, 15*). Approximately 1 mg tissue was thawed in 500 µL relaxing buffer (mM: 7.8 ATP, 20 phosphocreatine, 20 imidazole, 4 EGTA, 12 Mg-propionate, 97.6 K-propionate; pH 7.0) containing 5 µL of 1X protease inhibitor cocktail (G6521, Promega). Tissue was mechanically disrupted using a T10 basic Ultra-Turrax® disperser (IKA, Staufen, Germany) by mixing on setting 3 for 10 s, resting on ice for 2 min and then repeating. Cells were then centrifuged for 3 min at 200 x g at 4ºC, supernatant removed, resuspended in 500 µL in relaxing buffer supplemented with 0.5% Triton X-100 and then incubated for 6 min on ice to demembranate the cells. The cell suspension was again centrifuged for 3 min at 200 x g at 4ºC and washed five times with relaxing solution to remove the Triton X-100.

Single demembranated cardiomyocytes were selected under an inverted microscope (Zeiss Axiovert 200, 40× objective, NA 0.75) and attached with shellac dissolved in 70% ethanol (120 mg/mL) to micromanipulator-positioned glass microneedles connected to a force transducer and a piezoelectric motor as part of the Myostretcher system (IonOptix, Westwood, MA, USA). Sarcomere length (SL) was monitored using a video camera and analysis software provided by the manufacturer, and independently controlled at stretch length by calibrating against pixel size of images taken. To measure passive force, single cardiomyocytes were exposed to equidistant stepwise length increases of 0.1 µm every 10 s (SL, 1.7-2.2 µm). All measurements were repeated three times and an average taken. A 10 min recovery period was left between every measurement. Cells were then exposed to 2 mM GSH and 0.5 mM diamide or 35 µM PDI in relaxing solution for 30 min at either slack length (SL 1.7 µm) or stretched length (SL 2.3 µm) and then the stepwise length increase protocol was repeated. Reductant DTT (5 mM) was applied to reverse oxidation. The maximum peak for each passive force measurement, at the end of each stretch step, was used for analysis. Experiments were conducted at room temperature. Viability of the cells was determined by the addition of calcium at the end of the experiment and only cells that produced active force in the presence of calcium were used for the analysis.

Double immunofluorescence microscopy

Mouse heart samples of ischemic and remote (non-ischemic) tissue were fixed in 4% paraformaldehyde, 15% saturated picric acid in 100 mM phosphate buffered saline overnight at 4°C, dehydrated via ascending ethanol series, embedded in paraffin, and analyzed as described (*16*). Briefly, thin sections (5-7 µm) were cut with an RM 2235 Leica microtome (Mannheim, Germany). Sections were rehydrated, blocked in peroxidase blocking buffer, and a citrate-EGTA antigen recovery protocol was performed. Slides were rinsed with phosphate buffer and then blocked with 5% bovine serum albumin including 0.5% Triton X-100 for 60 min. Subsequently, sections were incubated with primary antibodies overnight at 4°C, using GSH monoclonal anti-mouse (mouse monoclonal anti-glutathione antibody ([D8]; ab19534, Abcam; 1:100) and titin I-band polyclonal anti-rabbit (TTN-5, Myomedix, Mannheim, Germany, 1:400). Secondary antibodies were Cy3- or FITC-conjugated IgG (Rockland; 1:250), which were incubated overnight at 4°C. Stained samples were embedded in Mowiol supplemented with N-propyl-gallate for bleaching protection and analyzed. Immunofluorescence imaging was performed with a Leica SP8 confocal laser scanning microscope equipped with an HC PL Apo CS2 63x NA 1.4 oil immersion objective, using the manufacturer's software.

Photometric detection of aggregation

The recombinant Ig-domains (wt and cysteine mutants of $_{82}Ig_{83}$) were buffered using a G25 column in 6 M guanidine hydrochloride solution. Then, 1 µL of the guanidine hydrochloride with protein solution was mixed with 200 µL of aggregation buffer (30 mM Tris/HCl, 50 mM KCl) and the absorbance at 320 nm was immediately measured every 2 s for 30 min (*15*). The same procedure was performed in presence of a mixture of 2 mM GSH and 0.5 mM diamide. After measurements, samples were centrifuged for 10 min at 10,000 x g and the insoluble pellet and the soluble supernatant were applied separately to glycine SDS gels, to confirm changes in oxidation state, as described under "S-glutathionylation and phosphorylation of Ig-domains".

AFM single-molecule force spectroscopy

Single-molecule force spectroscopy experiments were conducted using a commercial AFM setup (Luigs and Neumann, Germany) and an 8-Ig-domain $_{82}Ig_{83}$ polyprotein (*12, 17*). All the buffered solutions used for AFM experiments were freshly degassed by vacuum prior to use. The polyproteins were diluted to a concentration of 0.1 mg/mL in HEPES buffer (mM: 10 HEPES, 150 NaCl, 1 EDTA, pH 7.0). Before adsorbing the polyprotein onto a gold-coated coverslip, the surface was cleaned by immersion in high-purity ethanol for 20 min, thoroughly rinsed with distilled water, and dried with compressed air. A small amount of the polyprotein solution (15 µL) was applied onto the gold surface and incubated for 15 min, allowing the formation of covalent thiolate bonds between the C-terminal cysteine residues of the construct and the metal surface while leaving the amino-terminus free for the cantilever tip during the experiment. Experiments were done at room temperature.

In a typical experimental cycle, a substrate molecule was successfully picked up after repeated attempts by pressing the cantilever tip against the surface. The piezo, following an automated procedure, subsequently retracted. Experiments typically consisted of a triple pulse (denature, quench and probe) mechanical protocol (*12*). If the attachment was maintained under a constant pulling force at a pre-specified set point (175 pN) to the end of denature pulse (usually, 2 s), the force was then rapidly quenched to 0 pN to allow the domains in the tethered substrate to refold for a certain period of time (usually, 5 s). At the end of the measurements, the probe pulse was applied (duration, 5 s) using the same set point force as in the denature pulse. Only the traces containing 7 or 8 unfolding steps during the denature pulse were included in the analysis. In the analysis of the tether length vs. time traces, we compared the number and size of the steps in the probe pulse to those in the denature pulse, and could thus estimate how many Ig domains were properly folded back to the native state during the quench period, or remained unfolded/misfolded.

Reference list, Supplementary Methods

1. A. G. Nickel, A. von Hardenberg, M. Hohl, J. R. Löffler, M. Kohlhaas, J. Becker, J. C. Reil, A. Kazakov, J. Bonnekoh, M. Stadelmaier, S. L. Puhl, M. Wagner, I. Bogeski, S. Cortassa, R. Kappl, B. Pasieka, M. Lafontaine, C. R. Lancaster, T. S. Blacker, A. R. Hall, M. R. Duchen, L. Kästner, P. Lipp, T. Zeller, C. Müller, A. Knopp, U. Laufs, M. Böhm, M. Hoth, C. Maack, Reversal of mitochondrial transhydrogenase causes oxidative stress in heart failure. *Cell Metab.*, **22**, 472-484 (2015).
2. K. Toischer, A. G. Rokita, B. Unsöld, W. Zhu, G. Kararigas, S. Sossalla, S. P. Reuter, A. Becker, N. Teucher, T. Seidler, C. Grebe, L. Preuss, S. N. Gupta, K. Schmidt, S. E. Lehnart, M. Krüger, W. A. Linke, J. Backs, V. Regitz-Zagrosek, K. Schäfer, L. J. Field, L. S. Maier, G. Hasenfuss, Differential cardiac remodeling in preload versus afterload. *Circulation*, **122**, 993-1003 (2010).

3. B. M. Mohamed, M. Schnelle, S. Khadjeh, D. Lbik, M. Herwig, W. A. Linke, G. Hasenfuss, K. Toischer, Molecular and structural transition mechanisms in long-term volume overload. *Eur J Heart Fail.*, **18**, 362-371 (2016).
4. D. Mazor, E. Golan, V. Philip, M. Katz, A. Jafe, Z. Ben-Zvi, N. Meyerstein, Red blood cell permeability to thiol compounds following oxidative stress. *Eur J Haematol.,* **57**, 241-246 (1996).
5. S. Kötter, M. Kazmierowska, C. Andresen, K. Bottermann, M. Grandoch, S. Gorressen, A. Heinen, J. M. Moll, J. Scheller, A. Gödecke, J. W. Fischer, J. P. Schmitt, M. Krüger, Titin-based cardiac myocyte stiffening contributes to early adaptive ventricular remodeling after myocardial infarction. *Circ Res.*, **119**, 1017-1029 (2016).
6. C. Lindemann, L. I. Leichert, Quantitative redox proteomics: the NOxICAT method. *Methods Mol Biol.*, **893**, 387-403 (2012).
7. K. Xie, C. Bunse, K. Marcus, L. I. Leichert, Quantifying changes in the bacterial thiol redox proteome during host-pathogen interaction. *Redox Biol.*, **21**, 101087 (2019).
8. C. Lindemann, N. Lupilova, A. Müller, B. Warscheid, H. E. Meyer, K. Kuhlmann, M. Eisenacher, L. I. Leichert, Redox proteomics uncovers peroxynitrite-sensitive proteins that help Escherichia coli to overcome nitrosative stress. *J Biol Chem.*, **288**, 19698-19714 (2013).
9. J. Cox, M. Mann, MaxQuant enables high peptide identification rates, individualized p.p.b.-range mass accuracies and proteome-wide protein quantification. *Nat Biotechnol.*, **26**, 1367-1372 (2009).
10. S. Tyanova, T. Temu, P. Sinitcyn, A. Carlson, M. Y. Hein, T. Geiger, M. Mann, J. Cox, The Perseus computational platform for comprehensive analysis of (prote)omics data. *Nat Methods,* **9**, 731-740 (2016).
11. Y. Perez-Riverol, A. Csordas, J. Bai, M. Bernal-Llinares, S. Hewapathirana, D. J. Kundu, A. Inuganti, J. Griss, G. Mayer, M. Eisenacher, E. Pérez, J. Uszkoreit, J. Pfeuffer, T. Sachsenberg, S. Yilmaz, S. Tiwary, J. Cox, E. Audain, M. Walzer, A. F. Jarnuczak, T. Ternent, A. Brazma, J. A. Vizcaíno, The PRIDE database and related tools and resources in 2019: improving support for quantification data. *Nucleic Acids Res.,* **47(D1)**, D442-D450 (2019).
12. J. Alegre-Cebollada, P. Kosuri, D. Giganti, E. Eckels, J. A. Rivas-Pardo, N. Hamdani, C. M. Warren, R. J. Solaro, W. A. Linke, J. M. Fernández, S-glutathionylation of cryptic cysteines enhances titin elasticity by blocking protein folding. *Cell*, **156**, 1235-1246 (2014).
13. E. C. Eckels, S. Haldar, R. Tapia-Rojo, J. A. Rivas-Pardo, J. M. Fernández, The mechanical power of titin folding. *Cell Rep.,* **27**, 1836-1847 (2019).
14. C. G. Dos Remedios, S. P. Lal, A. Li, J. McNamara, A. Keogh, P. S. Macdonald, R. Cooke, E. Ehler, R. Knöll, S. B. Marston, J. Stelzer, H. Granzier, C. Bezzina, S. van Dijk, F. De Man, G. J. M. Stienen, J. Odeberg, F. Pontén, W. A. Linke, J. van der Velden, The Sydney Heart Bank: improving translational research while eliminating or reducing the use of animal models of human heart disease. *Biophys Rev.*, **9**, 431-441 (2017).
15. S. Kötter, A. Unger, N. Hamdani, P. Lang, M. Vorgerd, L. Nagel-Steger, W. A. Linke, Human myocytes are protected from titin aggregation-induced stiffening by small heat shock proteins. *J Cell Biol.*, **204**, 187-202 (2014).
16. A. Unger, L. Beckendorf, P. Böhme, R. Kley, M. von Frieling-Salewsky, H. Lochmüller, R. Schröder, D. O. Fürst, M. Vorgerd, W. A. Linke, Translocation of molecular chaperones to the titin springs is common in skeletal myopathy patients and affects sarcomere function. *Acta Neuropathol Commun.*, **5**, 72 (2017).
17. I. Popa, P. Kosuri, J. Alegre-Cebollada, S. Garcia-Manyes, J. M. Fernandez, Force dependency of biochemical reactions measured by single-molecule force-clamp spectroscopy. *Nat Protoc.*, **8**, 1261-1276 (2013).

Supplementary Tables

Table S1: Descriptive statistics for the volcano plots (Figures 1A-D and 2A, B) relative oxidation change values generated from peptides detected using the ICAT labeling MS analysis.

	Whole proteome						General titin					
	Number of peptides detected in each area on the volcano plot						Number of peptides detected in each area on the volcano plot					
Sample	Total peptides detected	Peptides with log2 oxidation change <-0.58	Peptides with log2 oxidation change between -0.58 & 0.58	Peptides with log2 oxidation change >0.58	Mean relative oxidation change	SEM	Total titin peptides detected	Peptides with log2 oxidation change <-0.58	Peptides with log2 oxidation change between -0.58 & 0.58	Peptides with log2 oxidation change >0.58	Mean relative oxidation change	SEM
H_2O_2 vs CTRL	1327	96	1180	51	4%	1%	125	1	75	49	49%	5%
H_2O_2 vs DTT	1338	178	990	170	7%	1%	117	5	83	29	23%	4%
Increased afterload vs CTRL	1214	55	1074	85	6%	1%	133	2	89	42	29%	3%
Increase preload vs CTRL	1234	33	1167	34	2.2%	0.6%	127	8	113	6	0.6%	3%
Skeletal non-stretch oxidized vs non-stretch CTRL	733	84	547	102	11%	2%	118	35	61	22	-0.6%	5%
Skeletal stretch oxidized vs non-stretch oxidized	686	75	528	83	6%	2%	127	29	76	22	9%	4%

Table S2: Descriptive statistics for the relative oxidation changes in titin shown in the colored bar graphs (Figures 1A-D and 2A, B) generated from peptides detected using the ICAT labeling MS analysis.

	Mean relative titin oxidation change					Mean relative I-band titin oxidation change				
Sample	I-band	SEM	A-Band	SEM	P value (Mann-Whitney)	Proximal	SEM	Distal	SEM	P value (Mann-Whitney)
H_2O_2 vs CTRL	58%	9%	45%	6%	0.11	54%	15%	61%	13%	0.75
H_2O_2 vs DTT	17%	9%	26%	4%	0.15	14%	16%	24%	12%	0.49
Increased afterload vs CTRL	25%	6%	28%	3%	0.63	16%	10%	23%	6%	0.44
Increased preload vs CTRL	17%	8%	-8%	2%	<0.0001	1%	5%	31%	14%	<0.04
Skeletal non-stretch oxidized vs non-stretch CTRL	-11%	6%	12%	8%	0.12	-24%	5%	14%	15%	0.0073
Skeletal stretch oxidized vs non-stretch oxidized	29%	6%	-11%	6%	<0.0001	26%	6%	34%	11%	0.77

Note: We have compared the level of relative oxidation change in the tandem-Ig domains of the proximal and the distal I-band titin region, excluding both the N2A and N2B elements.

Table S3. Amino acid sequences of Ig-domains from distal I-band titin expressed recombinantly and common S-glutathionylation sequences.

Protein	Sequences
$_{82}$Ig$_{83}$ (CC) (11.4 kDa)	NH_2-GSPEFPGRPRLIKVEKPLYGVEVFVGETAHFEIELSEPDVHGQWKLKGQPLTA SPDCEIIEDGKKHILILHN**C**QLGMTGEVSFQAANAKSAANLKVKVGGRIVTD-COOH
Ig84 (11.0 kDa)	NH_2-GSEGDPYFTGKLQDYTGVEKDEVILQCEISKADAPVKWFKDGKEIKPSKNAVI KADGKKRMLILKKALKSDIGQYTCDCGTDKTSGKLDIEGIPGSTRAAAS-COOH
$_{84}$Ig$_{85}$ (11.7 kDa)	NH_2-GSPEFREIKLVRPLHSVEVMETETARFETEISEDDIHANWKLKGEALLQTPDC EIKEEGKIHSLVLHNCRLDQTGGVDFQAANVKSSAHLRVKLGRLERPHRD-COOH
3-Ig-domain construct (31.6 kDa)	NH_2-GSPEFPGRQADPYFTVKLHDKTAVEKDEITLKCEVSKDVPVKWFKDGEEIVPS PKYSIKADGLRRILKIKKADLKDKGEYVCDCGTDKTKANVTVEARLIKVEKPLYGVE VFVGETAHFEIELSEPDVHGQWKLKGQPLTASPDCEIIEDGKKHILILHNCQLGMTG EVSFQAANAKSAANLKVKELPLIFITPLSDVKVFEKDEAKFECEVSREPKTFRWLKG TQEITGDDRFELIKDGTKHSMVIKSAAFEDEAKYMFEAEDKHTSGKLIIEVGGRIVTD -COOH

In red are the positions of the oxidized cysteines frequently detected by MS analysis in our various heart models (these are likely S-glutathionylation sites). In blue are the positions of the amino acids characteristic of the local environment of an S-glutathionylation site. The domain nomenclature follows UniProtKB entry for the mouse titin consensus sequence (A2ASS6).

Table S4: Recombinant Ig-domain $_{82}$Ig$_{83}$ with cysteine and phosphosite mutants generated.

**$_{82}$Ig$_{83}$ mutants**	**Sequences**
$_{82}$Ig$_{83}$ (CA) (9.7 kDa)	NH_2-RLIKVEKPLYGVEVFVGETAHFEIELSEPDVHGQWKLKGQPLTASPDCEIIE DGKKHILILHNAQLGMTGEVSFQAANAKSAANLKVK-COOH
$_{82}$Ig$_{83}$ (AC) (9.7 kDa)	NH_2-RLIKVEKPLYGVEVFVGETAHFEIELSEPDVHGQWKLKGQPLTASPDAEIIE DGKKHILILHNCQLGMTGEVSFQAANAKSAANLKVK-COOH
$_{82}$Ig$_{83}$ (AA) (9.9 kDa)	NH_2-GSLIEVEKPLYGVEVFVGETAHFEIELSEPDVHGQWKLKGQPLAASPDAEII EDGKKHILILHNAQLGMTGEVSFQAANTKSAANLKVKEL-COOH
$_{82}$Ig$_{83}$ (Phos-0) (9.5 kDa)	NH_2-RLIKVEKPLAGVEVFVGEAAHFEIELAEPDVHGQWKLKGQPLAAAPDCEIIE DGKKHILILHNCQLGMAGEVAFQAANAKAAANLKVK-COOH
$_{82}$Ig$_{83}$ (Phos-1) (9.5 kDa)	NH_2-RLIKVEKPLYGVEVFVGEAAHFEIELAEPDVHGQWKLKGQPLAAAPDCEIIE DGKKHILILHNCQLGMAGEVAFQAANAKAAANLKVK-COOH
$_{82}$Ig$_{83}$ (Phos-2) (9.5 kDa)	NH_2-RLIKVEKPLAGVEVFVGETAHFEIELAEPDVHGQWKLKGQPLAAAPDCEIIE DGKKHILILHNCQLGMAGEVAFQAANAKAAANLKVK-COOH
$_{82}$Ig$_{83}$ (Phos-3) (9.5 kDa)	NH_2-RLIKVEKPLAGVEVFVGEAAHFEIELSEPDVHGQWKLKGQPLAAAPDCEIIE DGKKHILILHNCQLGMAGEVAFQAANAKAAANLKVK-COOH
$_{82}$Ig$_{83}$ (Phos-4) (9.5 kDa)	NH_2-RLIKVEKPLAGVEVFVGEAAHFEIELAEPDVHGQWKLKGQPLTAAPDCEIIE DGKKHILILHNCQLGMAGEVAFQAANAKAAANLKVK-COOH
$_{82}$Ig$_{83}$ (Phos-5) (9.5 kDa)	NH_2-RLIKVEKPLAGVEVFVGEAAHFEIELAEPDVHGQWKLKGQPLAASPDCEIIE DGKKHILILHNCQLGMAGEVAFQAANAKAAANLKVK-COOH
$_{82}$Ig$_{83}$ (Phos-6) (9.5 kDa)	NH_2-RLIKVEKPLAGVEVFVGEAAHFEIELAEPDVHGQWKLKGQPLAAAPDCEIIE DGKKHILILHNCQLGMTGEVAFQAANAKAAANLKVK-COOH
$_{82}$Ig$_{83}$ (Phos-7) (9.5 kDa)	NH_2-RLIKVEKPLAGVEVFVGEAAHFEIELAEPDVHGQWKLKGQPLAAAPDCEIIE DGKKHILILHNCQLGMAGEVSFQAANAKAAANLKVK-COOH
$_{82}$Ig$_{83}$ (Phos-8) (9.5 kDa)	NH_2-RLIKVEKPLAGVEVFVGEAAHFEIELAEPDVHGQWKLKGQPLAAAPDCEIIE DGKKHILILHNCQLGMAGEVAFQAANAKSAANLKVK-COOH

Mutated sites are shown in red. In blue are the positions of the cysteines or the potential phosphorylation site remaining in a construct. The domain nomenclature follows UniProtKB entry for the mouse titin consensus sequence (A2ASS6).

Supplementary Figures

Figure S1

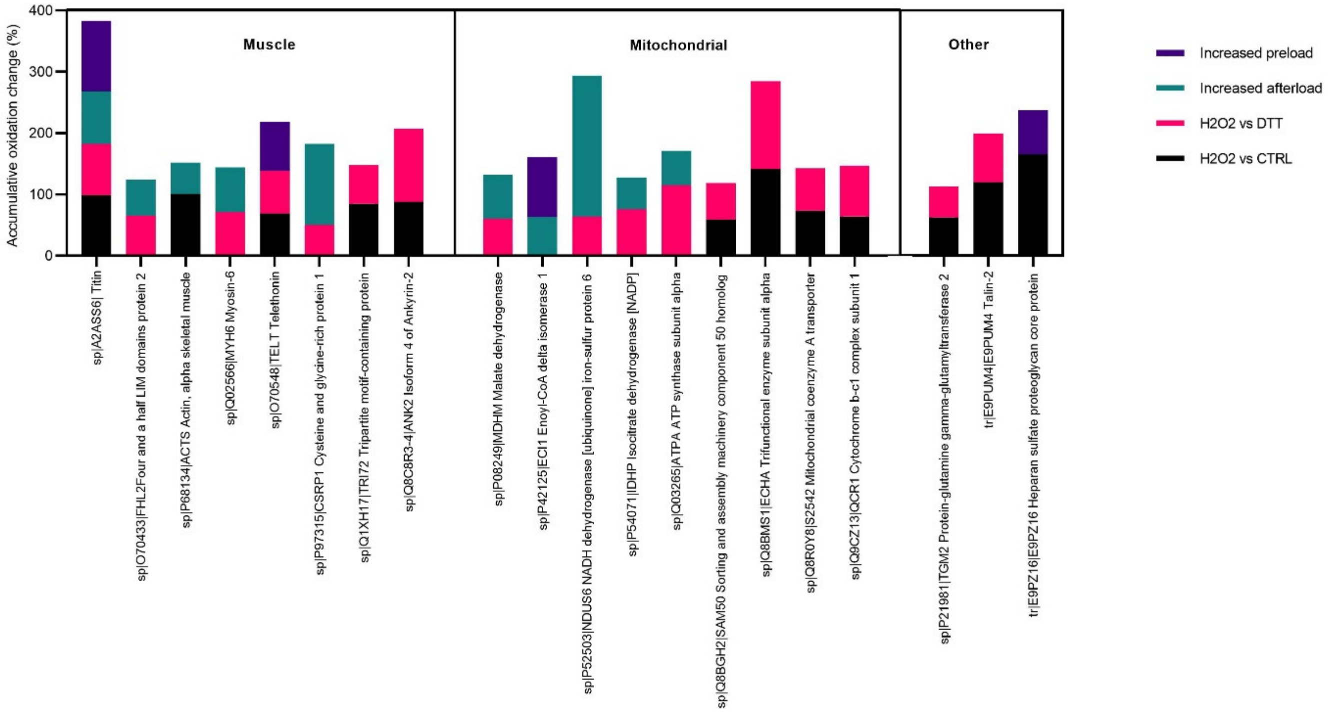

Fig. S1. Frequently oxidized peptides in different mouse heart models. The accumulative percentage oxidation change in the peptides detected using the ICAT labeling MS analysis across the four different heart models (H_2O_2 vs CTRL, H_2O_2 vs DTT, increased afterload vs CTRL and increase preload vs CTRL; see Supplementary Methods). Only peptides that showed over a 50% increase in oxidation and were detected in more than one heart model are included. The peptide nomenclature follows UniProtKB entry for the mouse (UP000000589).

Figure S2

A *Ex vivo* oxidative stress

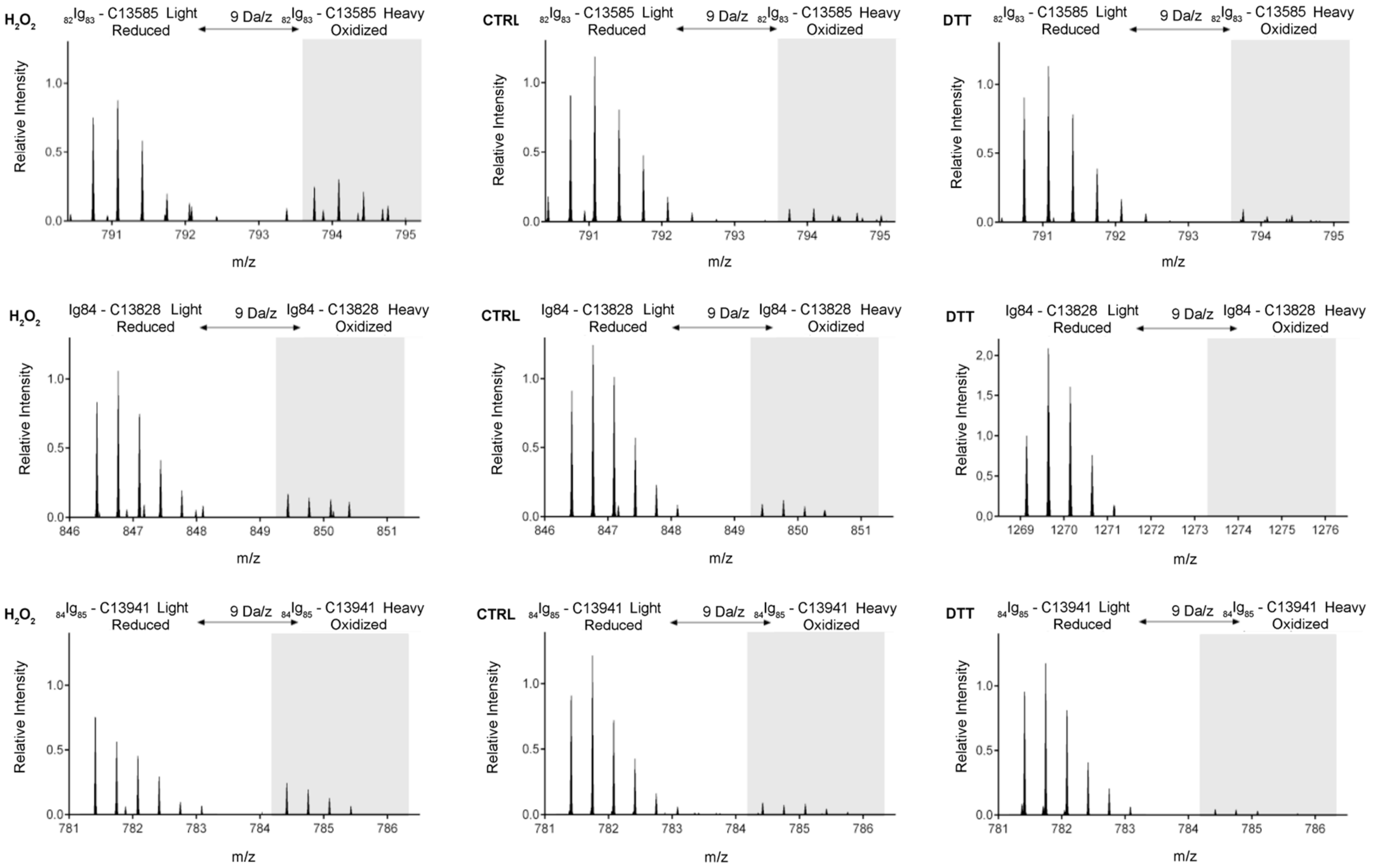

B *Ex vivo* increased afterload

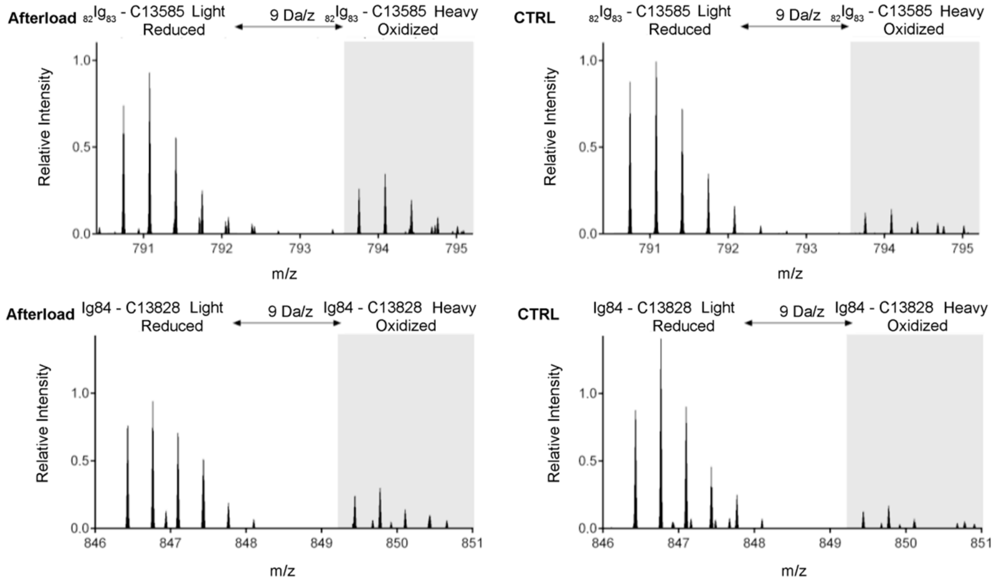

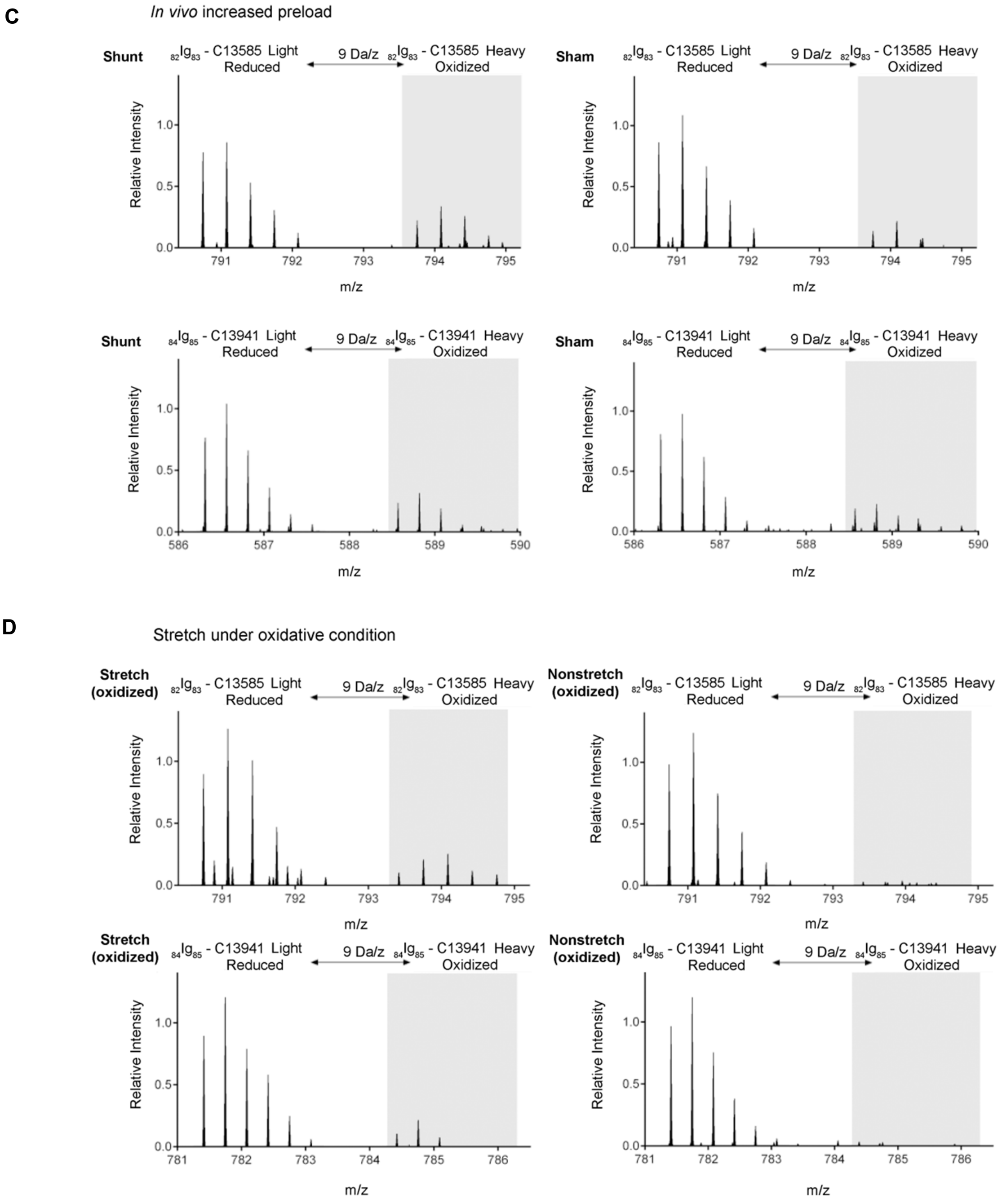

Fig. S2. Representative MS spectra of oxidized titin peptides regularly detected in the distal I-band Ig region. (**A**) MS spectra of cysteines after *ex vivo* oxidative stress induced with Langendorff perfusion of H_2O_2, CTRL or DTT; (**B**) *ex vivo* increased afterload and CTRL; (**C**) *in vivo* shunt vs. sham; (**D**) stretched and nonstretched skeletal muscle under oxidative conditions. Spectra of cysteine C13585 ($_{82}$Ig$_{83}$; NH_2 LKGEPLTASPDCEIIEDGKK-COOH) with a charge of +3 and a monoisotopic peak for the light ICAT of 790.74, C13828 (Ig84; NH_2 LQDYTGVEKDEVILQCEISK-COOH) with a charge of +3 and a monoisotopic peak at 846.43 and a charge of +2 and an monoisotopic peak of 1269.15 for DTT, C13941 ($_{84}$Ig$_{85}$; NH_2 LKGEALLQTPECEIKEEGK-COOH) with a charge of +3 and a monoisotopic peak of 781.41 or +4 and a monoisotopic peak of 586.31 in the shunt and sham models. The peaks of the ICAT pairs are normalized to the sum of the intensities of the monoisotopic peaks of the light and heavy ICAT forms.
